# Metabolic Programming Drives Protective and Inflammatory Monocyte Fates in Viral Encephalitis

**DOI:** 10.1002/advs.202505844

**Published:** 2025-07-14

**Authors:** Claire L. Wishart, Alanna G. Spiteri, Jian Tan, Laurence Macia, Nicholas J. C. King

**Affiliations:** ^1^ Infection, Immunity, Inflammation Research Theme School of Medical Sciences Faculty of Medicine and Health The University of Sydney Sydney NSW NSW 2006 Australia; ^2^ Charles Perkins Centre The University of Sydney Sydney NSW NSW 2006 Australia; ^3^ Chronic Diseases Research Theme School of Medical Sciences Faculty of Medicine and Health The University of Sydney Sydney NSW NSW 2006 Australia; ^4^ Sydney Institute for Infectious Diseases Faculty of Medicine and Health The University of Sydney Sydney NSW NSW 2006 Australia; ^5^ The University of Sydney Nano Institute Faculty of Science The University of Sydney Sydney NSW NSW 2006 Australia; ^6^ Honorary Professor of Immunopathology Foundation Academic Director Sydney Cytometry School of Medical Sciences Faculty of Medicine and Health University of Sydney

**Keywords:** glycolysis, Immunometabolism, monocyte‐derived cells, monocytes, viral encephalitis, West Nile virus

## Abstract

Infiltrating monocytes can exert both protective and pathogenic effects during central nervous system (CNS) inflammation. However, the metabolic mechanisms that govern these divergent roles remain poorly understood, limiting opportunities for therapeutic intervention. Single‐cell RNA‐sequencing and metabolic flow analysis of brain and bone marrow (BM) is used to map the metabolic signatures of monocyte‐derived cells (MCs) during lethal West Nile virus encephalitis. Trajectory analysis shows that BM monocytes progress through three metabolic profiles before migrating to the brain and differentiating into a pro‐inflammatory HIF1‐α⁺ MC population. This population further diverges into an inflammatory, iNOS⁺ MC subset with high glycolysis and amino acid metabolism, and a protective, glycolytically quiescent, antigen‐presenting MC subset. Daily in vivo glycolysis inhibition reduces neuroinflammation and disease signs without increasing viral load. This effect does not reflect a broad reduction in myelopoiesis but rather a selective decrease in iNOS⁺ MC migration, revealing distinct glycolytic dependencies among MC subsets. HIF1‐α activity remains independent of glycolysis, enabling functional differentiation of antigen‐presenting MCs without impairing antiviral responses by cervical lymph node T cells. This study identifies key metabolic drivers of MC function in viral CNS disease, in which selective metabolic reprogramming reduces severe neuroinflammation, demonstrating a promising therapeutic strategy.

## Graphical abstract

Bone marrow monocytes infiltrate the West Nile virus‐infected brain and differentiate into HIF1‐α⁺ monocyte‐derived cells (MCs) that diverge into protective, antigen‐presenting or inflammatory, iNOS⁺ MCs with distinct metabolic programs and functions. Glycolysis inhibition selectively depletes glycolysis‐dependent iNOS⁺ MCs without impairing antigen presentation or antiviral T cell responses, offering a targeted metabolic strategy to modulate neuroinflammation without compromising immunity.

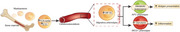

## 1 Introduction

Monocytes play a pivotal role in both inflammation and maintenance of tissue homeostasis. In the central nervous system (CNS), they replenish a small proportion of tissue‐resident macrophages in the dura mater and choroid plexus,^[^ ^1^, ^2^ ^]^ and while typically constrained by the blood‐brain barrier, readily infiltrate the CNS during inflammation.^[^ ^3^, ^4^ ^]^ Monocyte‐derived effector cells (MCs), such as dendritic cells and macrophages, play crucial roles in CNS disorders. Their diverse functions include innate host defence, (e,g., phagocytosis of pathogens and tissue debris), initiation of adaptive defences (antigen presentation) and tissue repair. However, the precise role of cellular metabolism in shaping these functions in the context of CNS disease remains poorly defined.

MC metabolism tends to be dichotomized simplistically into inflammatory (M1) and regulatory (M2) profiles, which are predominantly studied in vitro.^[^ ^5^ ^]^ In this classification, M2 MCs have regulatory functions that rely on an oxygen‐dependent pathway supporting slower, but more efficient, ATP generation, while M1 MCs exert pro‐inflammatory functions and rely on glycolysis to meet their high energy demands.^[^ ^6^ ^]^ Various M1 stimuli, such as interferon (IFN)‐γ, lipopolysaccharide (LPS), tumour necrosis factor, viruses, and granulocyte‐macrophage colony‐stimulating factor, are characterized by their proinflammatory effects, yet they induce significantly different phenotypes and metabolic responses. This suggests a more complex and nuanced metabolic framework underpinning inflammatory functions. Supporting this, disease environments comprised of diverse inflammatory stimuli give rise to metabolic states tailored to cellular functions.^[^ ^7^, ^8^ ^]^ Such functions include nitric oxide (NO) production, type I interferon responses,^[^ ^9^, ^10^, ^11^ ^]^ cytokine synthesis,^[^ ^12^, ^13^ ^]^ antigen presentation,^[^ ^14^ ^]^ phagocytosis,^[^ ^13^, ^15^, ^16^ ^]^ and migration into inflamed tissues.^[^ ^17^, ^18^ ^]^

West Nile virus (WNV) is a mosquito‐borne, neurotropic flavivirus and one of the most important causative agents of human viral encephalitis worldwide.^[^ ^19^ ^]^ In its neuroinvasive phase, WNV can cause severe encephalitis, the pathogenesis of which is driven by infiltration of M1‐like MCs into the CNS. Murine models clearly show that monocytes recruited from the bone marrow (BM) migrate to the brain in a CC motif chemokine ligand 2 (CCL2)‐dependent manner, diapedesing into the CNS via Ly6C and activated very late antigen‐4 (VLA‐4) expressed on the monocyte surface. Once in the CNS they mediate immune pathology by the sustained production of NO.^[^ ^20^, ^21^, ^22^ ^]^ Strategies that block the entry of inflammatory monocytes into the brain, such as by CCL2, VLA‐4 or Ly6C antibody blockade or immune modifying particles, or that attenuate their inflammatory response, such as inhibition of NO production using aminoguanidine hemisulphate, have been shown to improve survival of infected mice markedly without affecting viral load,^[^ ^20^, ^21^, ^22^, ^23^, ^24^ ^]^ thus emphasizing the role of MCs in causing the inflammatory damage observed in WNV infection. Notably, these cells also adopt an antigen‐presenting phenotype^[^ ^24^ ^]^ and may indirectly aid in viral clearance by promoting an effective T cell response. This effect is more pronounced in peripheral inoculation models, where monocytes exhibit a more protective function,^[^ ^25^ ^]^ in contrast to models in which WNV infection is confined to the CNS, in which viral spread and disease progression is more accelerated. However, it is unclear whether these differential functions are reflected in and driven by distinct metabolic profiles.

Numerous tools exist for the easy assessment of metabolism *ex vivo*. However, traditional methods that measure metabolic respiration in bulk or across whole populations fail to capture the increasingly recognized metabolic diversity of MC populations in the context of disease. To address this, recent technological advancements have enabled single‐cell analysis of metabolism through two primary methods. The first uses single‐cell RNA sequencing (scRNA‐seq) for broad analysis of gene expression related to metabolic changes. The second combines cytometric techniques with the detection of key metabolic enzymes, transporters, and transcription factors as a proxy measurement of the metabolic profile.^[^ ^26^, ^27^, ^28^, ^29^ ^]^ An additional cytometric approach, single cell energetic metabolism by profiling translation inhibition (SCENITH), combines metabolic inhibitors and protein synthesis quantification to assess the metabolic dependencies of immune cells at the single‐cell level.^[^ ^30^ ^]^ The latter approaches overcome the limitations of inferring function from gene expression alone. Nevertheless, comprehensive integration of gene and protein data at the single‐cell level is ultimately required to fully elucidate and map the cellular metabolic processes that lead to disease resolution or exacerbation.

In this study, we identify tissue‐ and time‐specific metabolic changes in response to severe flaviviral infection in the CNS, using both scRNA‐seq and flow‐based metabolic analysis. In particular, we show that MCs with a high glycolytic phenotype exhibited an inflammatory M1‐like phenotype that distinguished them from antigen‐presenting and other MC populations. Targeting glycolysis as an anti‐inflammatory strategy preferentially affected M1‐like cells while sparing antigen‐presenting populations, resulting in amelioration of disease without increasing viral burden. This selective targeting emphasizes the potential of metabolic interventions in managing the intricate temporal pathogenesis of complex CNS disorders.

## 2 Results

### 2.1 MCs Adopt Distinct Metabolic and Functional Profiles in CNS Infection

Since BM‐derived MCs infiltrating the CNS contribute significantly to immunopathology in WNV encephalitis, we sought to identify the specific metabolic pathways associated with the differentiation and development of these pathogenic responses. Monocyte‐derived cells (MCs) were sorted flow cytometrically from the brain at 5 and 7 days post‐infection (dpi) and identified as Ly6G^−^, CD49^hi^, P2RY12^lo^, NK1.1^−^, CD3e^−^, CD11b^+^, CD64^+^ and CX3CR1^+^, capturing the entire population of Ly6C^hi^ and Ly6C^lo^ MCs at 5 and 7 dpi.^[^ ^24^, ^31^ ^]^ Mature monocytes from the BM at 7 dpi were identified as CD45.2^+^, Ly6G^−^, CD48^hi^, NK1.1^−^, CD3e^−^, B220^−^, CD11b^+^, CD117^−^, CD115^hi^, as previously described.^[^ ^24^ ^]^ To ensure that brain MCs were not contaminated with the phenotypically similar microglia population, we separately sorted and barcoded microglia with a validated gating approach (defined as Ly6G⁻, CD49d^lo^, P2RY12^hi^, CD11b⁺, and CX3CR1⁺)^[^ ^31^, ^32^ ^]^ and confirmed their identity by differential expression of microglia‐specific genes and monocyte lineage genes (Figure S1, Supporting Information). Based on gating, barcoding, and transcriptional profiles, microglia were then excluded from downstream analyses.

**Figure 1:**
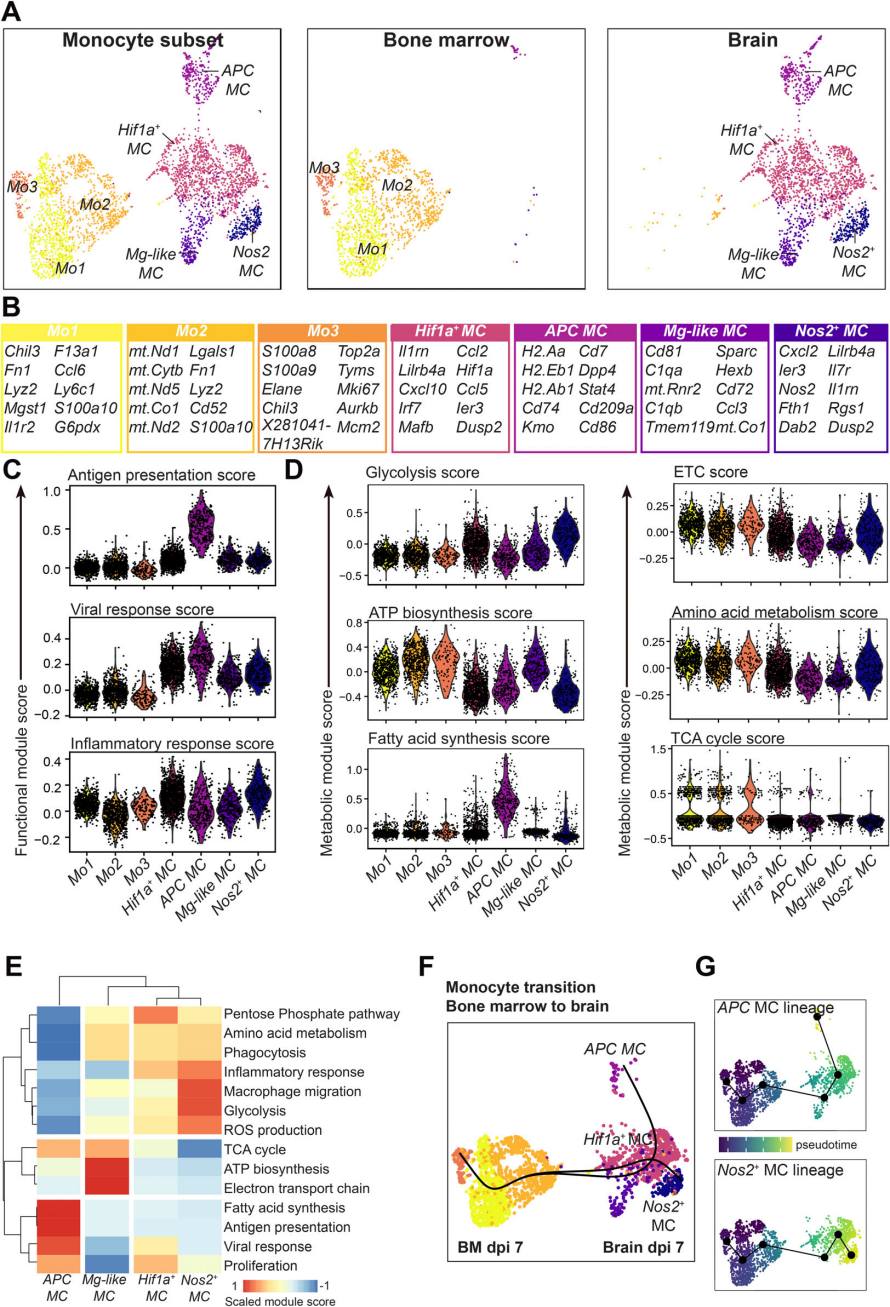
MCs adopt distinct metabolic and functional transcriptomic profiles in CNS infection. A) UMAPs of monocyte‐derived cells identified in WNV infection across the BM at 7 dpi and brain at 5 and 7 dpi. B) Top differentially expressed genes per cluster. Differentially expressed genes were defined as genes enriched in a cluster versus all other clusters. C,D) Module scores for functional (C) and metabolic (D) pathways across each of the identified subsets. E) Heatmap depicting the scaled average module scores for *APC*, *Mg‐like*, *Hif1a^+^*, and *Nos2^+^* MCs in the brain across selected metabolic and functional modules. F) Pseudotime trajectory analysis on BM and brain myeloid cells using Slingshot with *Mo3* set as the root (starting) subset. UMAP is colour‐coded by subsets shown in (A). G) Lineage trajectories from Slingshot analysis, 1) *Nos2* ^+^ *MC* and 2) *APC MC*. UMAPs are coloured by pseudotime. Data is from one experiment with two samples per group, with two mice pooled per sample (4 mice total per group).

**Figure 2:**
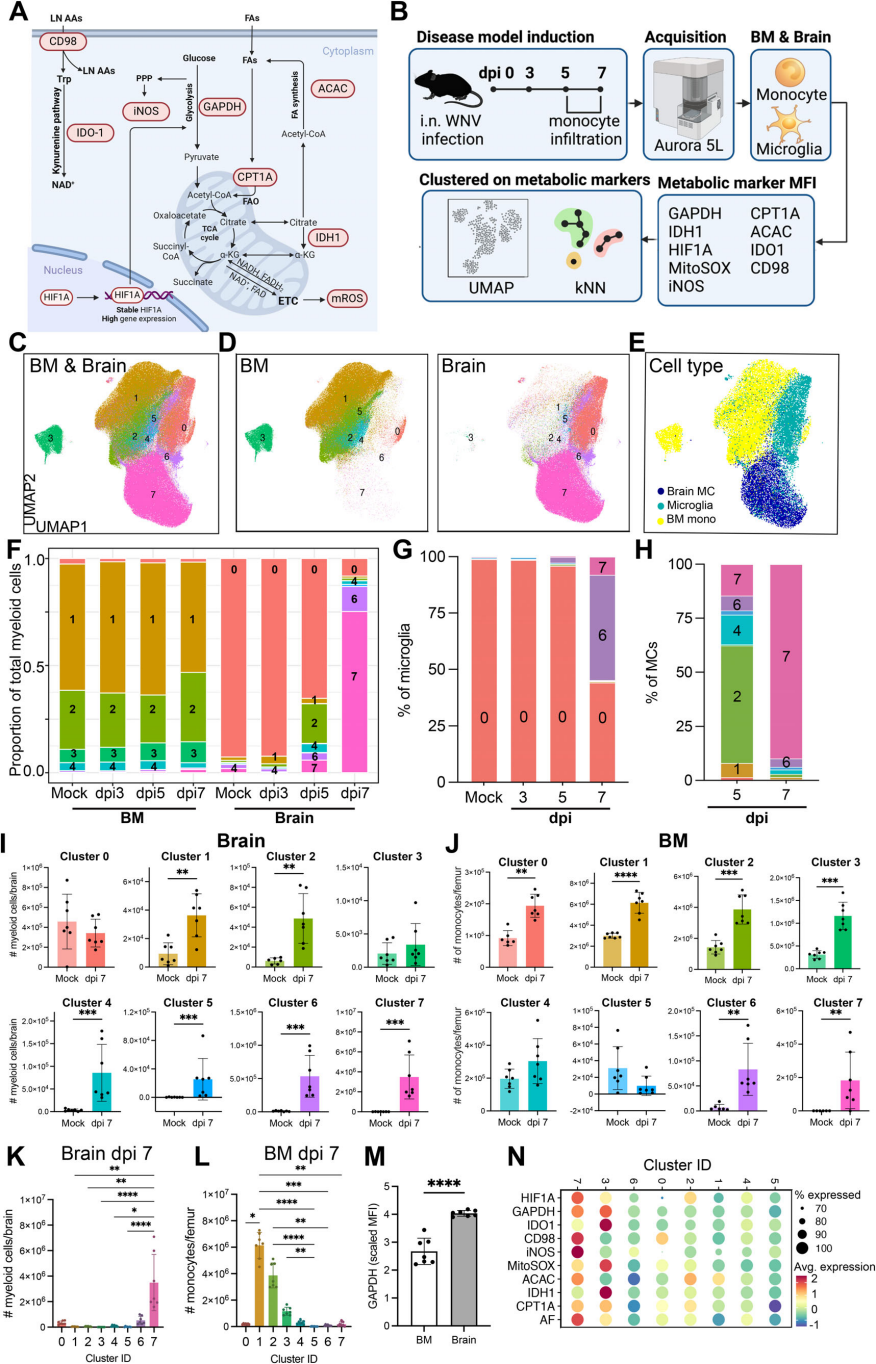
Metabolic heterogeneity of MCs in viral infection by spectral flow cytometry. A) Schematic of metabolic targets for flow‐based analysis. B) Experimental workflow. The bone marrow (BM) and brain were isolated from mock‐infected and WNV‐infected mice at 3, 5, or 7 dpi and stained with a metabolic panel. UMAP and k‐nearest neighbour clustering was performed on BM monocytes, brain microglia and brain MCs across time points based on arcsinh transformed metabolic protein MFI. C–E) UMAPs pseudocolored by metabolic cluster ID (C, D) and cell types (E) in the BM and brain. F–H) Proportion of each metabolic cluster comprising the total myeloid pool across organs and time points (F), or the total microglia (G), or the MC (H) population in the brain. I,J) Number of myeloid cells per brain (I) and monocytes per femur (J) in each cluster in mock‐infected and WNV‐infected mice at 7 dpi. K,L) Number of myeloid cells per brain (K) and monocytes per femur (L) in each cluster at 7 dpi. M) Arcsinh transformed median fluorescence intensity (MFI) of GAPDH in the BM and brain at 7 dpi. N) Dot plot heatmap depicting the MFI of each metabolic marker across the identified metabolic cluster at 7 dpi. Size of dot represents the percentage of cells in each cluster expressing the metabolic marker. Data is pooled from two independent experiments with 3–4 mice per group. Statistics done using Mann‐Whitney test (I, J, M) or Kruskal‐Wallis test with Dunn's test for multiple comparisons (K, L). * *p*<0.05, ** *p* <0.01, *** *p*<0.001, **** *p* <0.0001. Error bars are representative of mean ± SD.

Clustering on scRNA‐seq data from 3498 MCs and BM monocytes revealed four distinct states in the brain and three in the BM, respectively (**Figure** **1**A). Of the brain subsets, we identified 1) an “antigen‐presenting cell (*APC*)” population highly expressing genes involved in antigen presentation, including *H2‐Aa*, *H2‐Eb1*, *H2‐Ab1*, *Cd74*, and *Cd86* (Figure 1B), 2) a *Hif1a‐*expressing population, 3) a *Nos2*‐expressing population, which likely represented the nitric oxide (NO)‐producing population causing inflammatory damage in this model, and 4) a *“Microglia (Mg)‐like MC”* subset resembling microglia, due to its relative expression of microglia‐specific markers *Cd81*, *Sparc*, *Hexb*, and *Tmem119* (Figure 1B).^[^ ^31^, ^33^ ^]^ The term “Mg‐like” MCs, previously defined by our group, describes a population of infiltrating bone marrow–derived macrophages adopting a microglia‐like phenotype in the WNV‐infected brain,^[^ ^31^ ^]^ consistent with the population observed here. Previous work has shown that this population expresses microglia‐specific markers at lower levels than bona fide microglia, yet at higher levels than BM monocytes and other brain‐infiltrating MCs,^[^ ^31^ ^]^ suggesting it represents a distinct intermediate phenotype. Based on a combination of barcoding strategy and the expression of cell type‐specific genes (Figure S1, Supporting Information), we were able to accurately distinguish between microglia and MCs that may adopt a microglia‐like profile.

The functional status of these MC clusters was supported by manually querying several M1 functional programs, such as *antigen presentation*, *inflammatory response*, and *viral response* against gene modules from the Mouse Genome database^[^ ^34^ ^]^ (Figure 1C). A high module score represents the average expression of the genes in the module relative to a set number of randomly extracted control genes from the dataset. The *APC MC* cluster had a high antigen‐presenting score and viral response score, suggesting this population may be involved in indirectly contributing to viral clearance (Figure 1C). By contrast, *Hif1a^+^* and *Nos2^+^* MCs exhibited a higher inflammatory response score, supporting a potential inflammatory phenotype that contributes to inflammatory damage in WNV encephalitis (Figure 1C).^[^ ^21^ ^]^ All BM monocyte clusters (*Mo1*‐*Mo3*) displayed low M1 functional scores, supporting the notion that these cells are an undifferentiated monocyte state (Figure 1C).

As metabolism is coupled with the phenotype and functionality of MCs, we next scored genes involved in several metabolic pathways known to be important in M1‐ or M2‐like functions, including glycolysis, adenosine triphosphate (ATP) biosynthesis, fatty acid synthesis, electron transport chain (ETC), amino acid metabolism, and the tricarboxylic acid (TCA) cycle (Figure 1D). BM monocytes (*Mo1*, *Mo2*, and *Mo3*) and *Mg‐like MC* displayed higher expression of metabolic pathways related to homeostatic functions, including the TCA cycle, ATP biosynthesis, and ETC, but also displayed high amino acid metabolism scores (Figure 1D,E). Inflammatory *Hif1a^+^* and *Nos2^+^* MCs displayed higher glycolysis and amino acid metabolism scores with low TCA cycle scores (Figure 1D). Expression of these metabolic pathways clustered with typical inflammatory functions, including the *inflammatory response, reactive oxygen species production*, and *phagocytosis* (Figure 1E), supporting the notion that these *Hif1a^+^* and *Nos2^+^* cells adopt a typical M1‐like phenotype in WNV encephalitis. On the other hand, *APC MCs* were evidently less reliant on glycolysis and amino acid metabolism pathways, but exhibited higher fatty acid synthesis scores (Figure 1D,E). This suggests that there exists wide metabolic heterogeneity in typical M1‐like functions in CNS infection, including the inflammatory response and antigen presentation, which is likely obscured in bulk in vitro systems.

To understand the differentiation pathway of BM monocytes into specific metabolic states in the CNS, we employed trajectory analysis. This showed that BM monocytes progressed through 3 different metabolic profiles before they migrated to the brain, where they underwent a transition toward either *APC* or *Nos2^+^ MC* populations in the brain at disease endpoint (Figure 1F). Both lineages passed through the *Hif1a^+^ MC* population, implying that this population is a transitional state. The *Mg‐like MC* population, however, did not align with these pathways, indicating that it is a unique MC population (Figure 1F). It could be argued that the distinctness of this 4^th^ population raises the question of contamination by microglial cells. However, as shown in Figure S1 (Supporting Information), microglia were excluded based on a combination of barcoding strategy and the expression of cell type–specific genes. Moreover, our prior work has shown that monocytes circulating in the bloodstream can adopt a microglial‐like phenotype within the WNV‐infected brain, possibly due to prolonged exposure to the CNS milieu.^[^ ^31^ ^]^ During their differentiation into brain MCs, BM monocytes consistently downregulated genes related to ATP synthesis, the TCA cycle, ETC and pentose phosphate pathway, while upregulating genes related to glycolysis (Figure S2, Supporting Information). This indicates a significant metabolic shift as MCs transition from the BM to the infected brain, possibly through a shared *Hif1a* ^+^ intermediate, before diverging into functionally distinct pathways toward antigen‐presenting (*APC*) or NO‐producing (*Nos2* ^+^) phenotypes.

### 2.2 Application of MetFlow to CNS Infection Reveals Distinct Metabolic Changes in CNS Disease

Given the limitations in existing tools to study metabolism by gene expression alone, we adapted MetFlow^[^ ^28^ ^]^ to murine cells and used metabolic marker proteins with high relevance to myeloid cells during inflammation. This comprised 13 immune cell identification markers and 9 metabolic marker proteins, including rate‐limiting enzymes, signalling molecules, and transcription factors that have roles in glycolysis, the tricarboxylic acid cycle, hypoxia‐induced inflammation, amino acid transport, fatty acid synthesis and oxidation, the kynurenine pathway, NO production, and mitochondrial ROS production (**Figure** **2**A; **Table** **1**).

**Table 1:** Metabolic targets included in panel.

Metabolic target	Name	Pathway
*GAPDH*	Glyceraldehyde 3‐phosphate dehydrogenase	Glycolysis
*IDH1*	Isocitrate dehydrogenase 1	TCA cycle
*CPT1A*	Carnitine Palmitoyltransferase 1A	Fatty acid oxidation
*ACAC*	Acetyl‐CoA carboxylase	Fatty acid synthesis
*CD98*	CD98	Amino acid transport and metabolism
*HIF1‐α*	Hypoxia‐inducible factor 1 alpha	Metabolic regulation/signaling during hypoxia and inflammation
*iNOS*	Inducible nitric oxide synthase	Nitric oxide production, amino acid metabolism
MitoSOX	N/A	Free radical superoxide generation
IDO1	Indoleamine 2,3‐dioxygenase	Amino acid metabolism (kynurenine pathway)

To identify metabolic signatures independently of cell lineage and origin, we performed dimensionality reduction on whole brain and BM cell isolates and clustered on metabolic markers (Figure 2B). Using a validated gating strategy,^[^ ^31^ ^]^ resident microglia (Ly6G^−^, SSCA^lo^, NK1.1^−^, CD3e^−^, B220^−^, CD45^low–int^, CX3CR1^+^, Ly6C^lo^), brain‐infiltrating MCs (defined as Ly6G^−^, SSCA^lo^, NK1.1^−^, CD3e^−^, B220^−^, CD45^hi^, CX3CR1^low^, Ly6C^hi^) and BM monocytes (defined as Ly6G^−^, SSCA^lo^, NK1.1^−^, CD3e^−^, B220^−^, CD11c^lo^, MHC‐II^lo^, CD11b^+^, Ly6C^hi/lo^, CX3CR1^hi/lo^) clustered into 8 distinct metabolic states which varied in proportion across timepoints and were distinctly grouped by organ and lineage/differentiation status (Figure 2C–H), demonstrating a significant metabolic adaptation over the course of infection. Within the brain, four dominant MC populations were detected (clusters 2, 4, 6 & 7) (Figure 2F,H), while three predominant monocyte populations were found in the BM (Figure 2F). This distribution mirrors the populations revealed by single‐cell RNA sequencing (Figure 1), corroborating our findings across both techniques.

In the brain, cluster 0 was associated with microglia and was found in the highest proportions in the mock‐infected brain (Figure 2F,G). By 7 dpi, microglia predominantly transitioned into cluster 6 (Figure 2G). However, the relative proportion of microglia was significantly reduced due to the massive influx of infiltrating MCs in the brain, which exceed microglia by approximately tenfold at this timepoint (Figure 2F). Thus, cell numbers in clusters 1, 2, 4, 5, 6, and 7, predominantly comprising infiltrating MCs, were markedly increased in the brain, compared to mock‐infected mice (Figure 2I), with numbers in cluster 7 some 8‐fold greater than the next largest, cluster 6 (Figure 2K). In the BM, clusters 0, 1, 2, 3, 6 and 7 were numerically increased at 7 dpi, relative to mock‐infected mice (Figure 2J), however, clusters 1, 2, and 3 comprised the majority of BM monocytes at 7 dpi, both numerically and by proportion (Figure 2F,L). These findings demonstrate clear metabolic remodelling both at peripheral sites of monocyte myelopoiesis and inflammatory foci in response to infection.

Supporting our scRNA‐seq findings, GAPDH was upregulated in the brain, compared to the BM (Figure 2M), emphasizing the importance of glycolysis in the differentiation of MCs. This was most obvious in cluster 7 expressing high levels of iNOS (Figure 2N), likely denoting the pathogenic NO‐producing MCs implicated in immunopathology. Along with increased iNOS expression, this cluster exhibited elevated GAPDH, HIF1‐α, and CD98 levels (Figure 2N), reflecting the *Nos2^+^* and *Hif1a^+^* profiles identified via scRNA‐seq (Figure 1) and constituted the majority of myeloid cells in the brain, as mentioned above (Figure 2K). In contrast to cluster 7, clusters 6, 4, and 2, the next‐largest remaining clusters, displayed lower expression of iNOS, HIF1‐α and GAPDH (Figure 2N), suggesting a deviation from typical pro‐inflammatory metabolic pathways. Interestingly, however, cluster 2, which expresses higher HIF1‐α, MitoSOX, and fatty acid metabolism markers (Figure 2N), is initially present in the brain by 5 dpi, coinciding with significant monocyte infiltration (Figure 2F,H). While remaining significantly elevated compared to controls (Figure 2L), cluster 2 had declined in the brain by 7 dpi (Figure 2H), corresponding with the emergence of iNOS^+^ cluster 7, suggesting it may be a precursor to this subset in the brain at 5 dpi and paralleling the trajectory observed in subsets identified by scRNAseq (Figure 1F,G).

The metabolic diversity of monocytes in the BM additionally reflects an adaptation to infection. BM cluster 3 is the only BM cluster with high GAPDH expression (Figure 2N) and it expands at 7 dpi in the BM (Figure 2J,L), suggesting this cluster is a possible BM precursor for the pathogenic iNOS^+^ state observed in the brain at 7 dpi. This notion is further supported by the close clustering of these populations by expression of their metabolic proteins (Figure 2N).

While the developmental trajectory of metabolic clusters in the BM is unclear, it is possible that the metabolically quiescent cluster 1 (Figure 2F), which, although increasing its majority proportion by some 10% during the early phase of infection, was reduced by dpi 7, transits progressively toward the more metabolically active subsets, cluster 2 and 3, which may give rise to distinct MC subsets in the brain. These findings suggest that the metabolic conditioning of BM cells may prime MCs for distinct trajectories of differentiation prior to their infiltration into the inflamed brain.

### 2.3 MHC‐II^+^ and iNOS^+^ MCs have Distinct Metabolic Profiles

**Figure 3:**
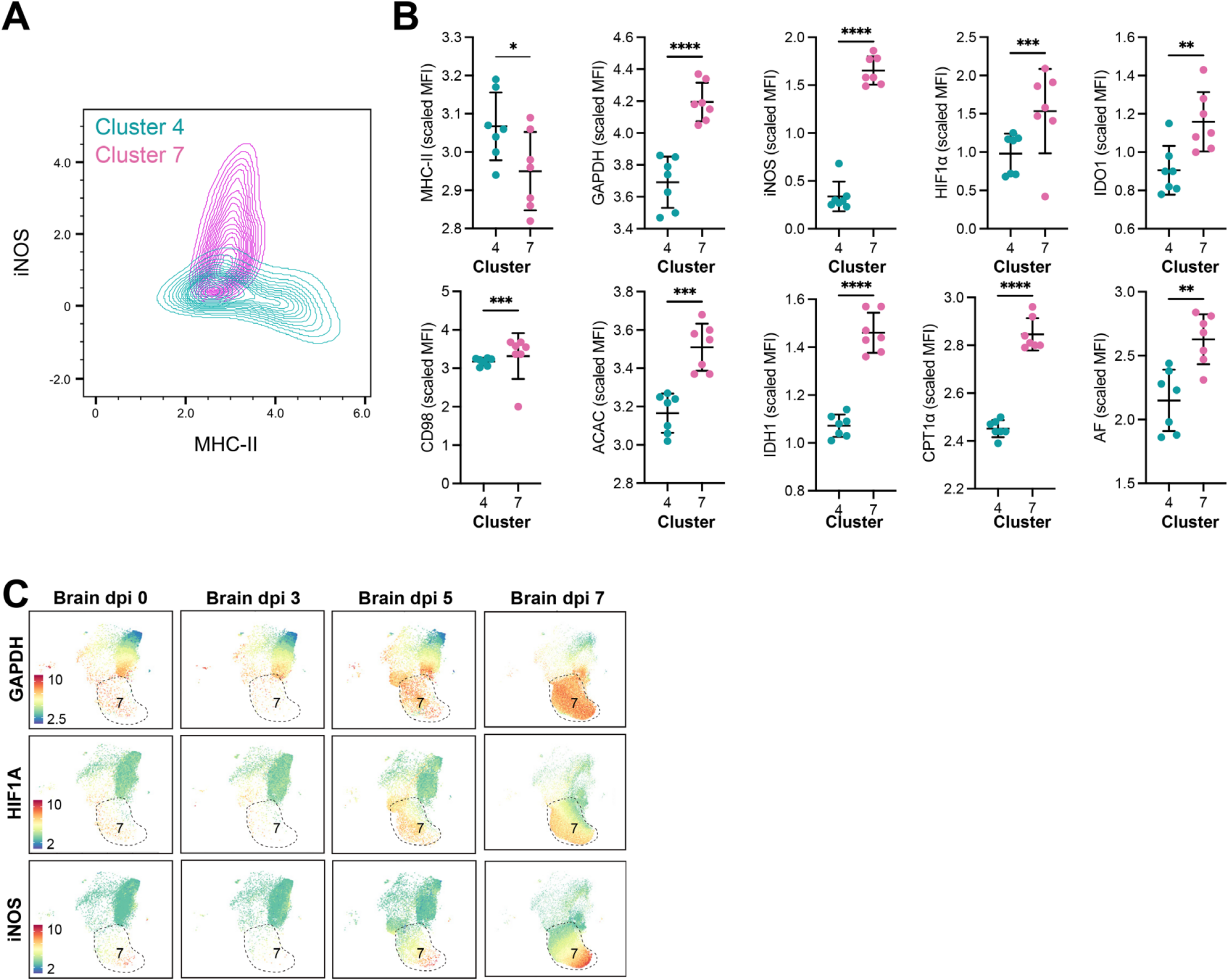
MHC‐II^+^ and iNOS^+^ MCs have distinct metabolic profiles in CNS viral infection. A) Contour plot depicting MHC‐II and iNOS expression of metabolic cluster 4 (blue) and 7 (pink). B) Arcsinh transformed MFI of selected functional and metabolic markers. C) UMAP showing the expression of GAPDH, HIF1‐α and iNOS in the brain in mock‐infected and WNV‐infected mice at 3, 5, and 7 dpi. Data is from two independent experiments with 3–4 mice per group. Statistics calculated with paired *t*‐test. * *p* < 0.05, ** *p* < 0.01, *** *p* < 0.001, **** *p* < 0.0001. Error bars are representative of mean ± SD.

As MCs in WNV may adopt both NO‐producing and antigen‐presenting phenotypes in the brain, we next aimed to determine if these functional profiles were reflected by metabolic differences. In Figure 1D, we previously provided a broad overview of metabolic protein expression across seven MC subsets defined by scRNA‐seq, identifying different metabolic profiles in *APC* and *Nos2^+^* MC subsets. However, given the well‐recognized disconnect between mRNA and protein expression, we reasoned that transcriptomic profiling alone may not fully capture the functional metabolic state of these subsets. To more clearly define the functional differences between NO‐producing (cluster 7) and antigen‐presenting (cluster 4) MC subsets, we therefore specifically examined their metabolic protein expression profiles in isolation. Cluster 7 and 4 were confirmed to be an NO‐producing and antigen‐presenting cell (APC) subset, respectively, based on their differential expression of MHC‐II and iNOS (**Figure** **3**A). Importantly, ≈80% of iNOS⁺ MCs were positive for DAF‐FM (Figure S3, Supporting Information), a fluorescent probe that stains for intracellular NO, supporting a direct link between iNOS expression and NO production. Intriguingly, compared to the NO‐producing cluster 7, the APC cluster 4 expressed all metabolic proteins at lower levels (Figure 3B). This observation suggests that the functional activity of cluster 7 might require synergism from multiple metabolic pathways to sustain this heightened inflammatory state, compared to that required for antigen presentation. Interestingly, these inflammatory iNOS^+^ cells displayed an increased expression of glycolysis‐related markers, such as GAPDH and HIF1‐α, compared to APC MCs (Figure 3B,C). These markers are known to be closely linked with glycolytic activity, suggesting that an APC MC phenotype is less reliant on glycolysis than the classical M1‐like phenotype associated with cluster 7. Collectively, these findings reinforce the notion that pathogenic, NO‐producing MCs utilize glycolysis differently from other MC subsets, such as antigen‐presenting cells, particularly in the context of virus‐induced neuroinflammation.

### 2.4 Glycolysis Inhibition is Protective in West Nile Virus Encephalitis

**Figure 4:**
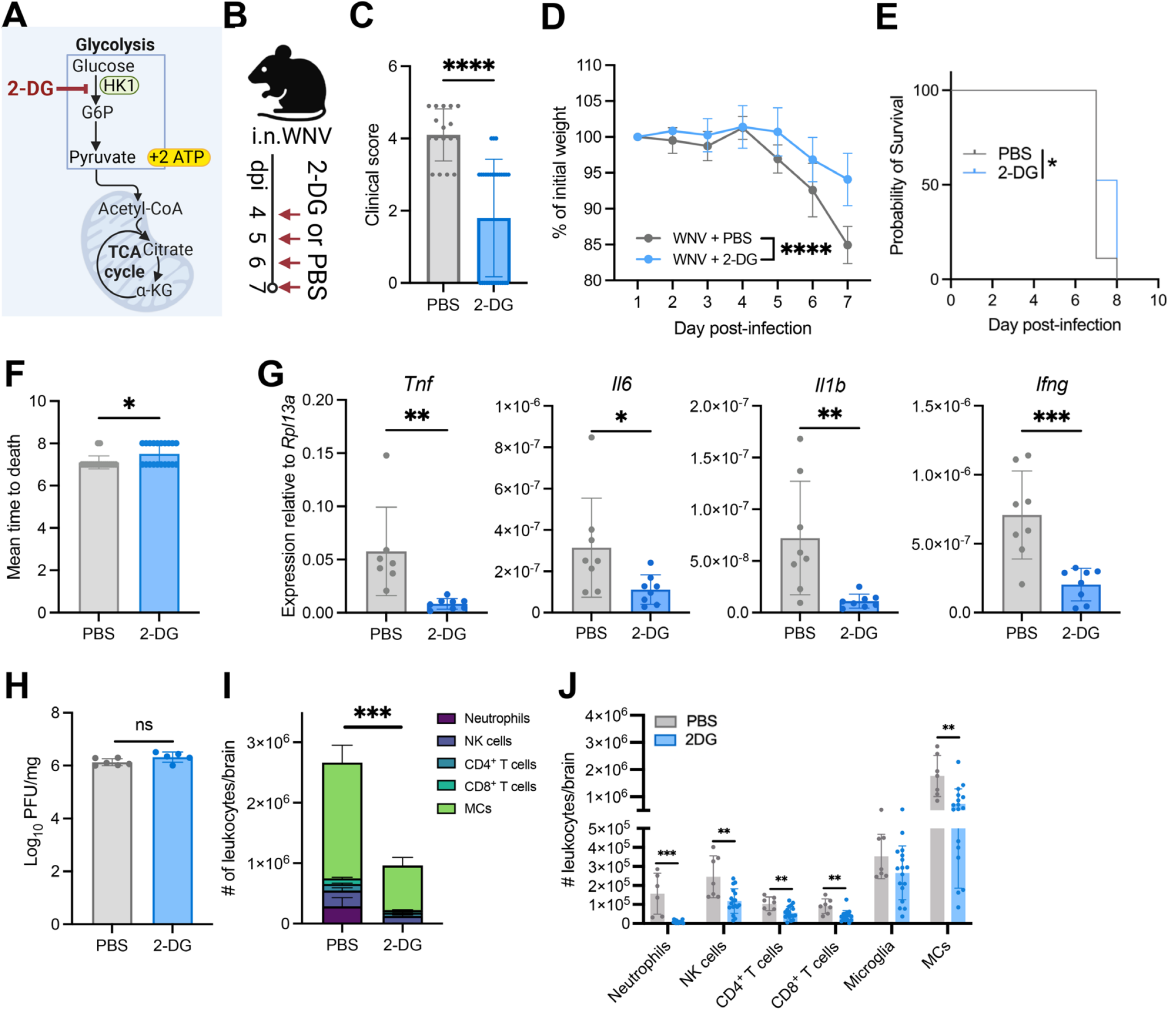
Glycolysis inhibition is protective in West Nile virus encephalitis. A) Schematic depicting 2‐DG mechanism of action. B) Schematic experimental design. WNV‐infected mice were treated with 2 g kg^−1^ of 2‐DG delivered i.p. daily from 4–7 dpi. C) Clinical scores of 2‐DG and PBS‐treated mice at 7 dpi. D) Weights of 2‐DG and PBS‐treated animals over the duration of infection shown as percentage of initial weight. E,F) Survival (E) and mean time to death (F) of WNV‐infected mice treated with 2‐DG or PBS (vehicle control). G). mRNA expression for selected pro‐inflammatory cytokines in the brains of PBS‐ and 2‐DG‐treated mice at dpi 7, as determined by qPCR. Expression was normalized to the housekeeping gene *Rpl13a*. H) WNV PFU in brains of 2‐DG‐ and PBS‐treated mice at 7 dpi. I) Stacked bar graph showing numbers of indicated CD45^+^ cell subsets in the brains of 2‐DG‐ and PBS‐treated mice at 7 dpi. J) Numbers of infiltrating and resident immune cell populations in the brain at 7 dpi. Data in (D) are pooled and representative of mean ± SD. Data is from one (E, F, G, H) or two (C, D, I, J) independent experiment(s) with at least five mice per group. Statistics calculated with Mann‐Whitney test (C, F, I, J), unpaired *t*‐test (D, G, H), or Kaplan‐Meier survival analysis (E). * *p* < 0.05, ** *p* < 0.01, *** *p* < 0.001, **** *p* < 0.0001. Error bars are representative of mean ± SD.

To determine whether pro‐inflammatory monocyte metabolism could be therapeutically targeted in lethal infection, we next treated mice with a glycolysis inhibitor, 2‐deoxy‐D‐glucose (2‐DG) (**Figure** **4**A), a nonmetabolizing glucose analogue and competitive inhibitor of hexokinase 1. 2‐DG was administered at a dose of 2 g kg^−1^ daily from 4 dpi to the disease endpoint at 7 dpi (Figure 4B). Remarkably, 2‐DG treatment led to a discernible clinical improvement, as indicated by lower disease severity scores at 7 dpi (Figure 4C) and reduced weight loss (Figure 4D), with a modest, but significant increase in mean time to death (Figure 4E,F). This was accompanied by significant reductions in inflammatory cytokine mRNA expression for *Tnf*, *Il6*, *Il1b*, and *Ifng*, indicating a global attenuation of neuroinflammation (Figure 4G). These improvements were not due to a reduction in viral load, as both 2‐DG and PBS‐treated mice exhibited comparable viral burdens in the brain at 7 dpi (Figure 4H), emphasizing that the effects of 2‐DG are likely mediated through modulation of the pathological immune response within the brain.

Notably, 2‐DG also reduced the overall neuroinflammatory infiltrate to ≈35% of that seen in control‐treated mice on day 7 post‐infection (Figure 4I). A significant reduction in cell numbers was observed in infiltrating neutrophils, natural killer cells, CD4^+^ and CD8^+^ T cells, and MCs in the brain, but not microglia (Figure 4J). This reduction with 2‐DG treatment was not due to changes in cell viability, as we observed only a minor decrease in viability (∼5%) among total leukocytes and no detectable change in the viability of total MCs following 2‐DG treatment (Figure S4, Supporting Information). Importantly, microglial numbers were not significantly changed following 2‐DG treatment, irrespective of inflammatory activation (Figure S4, Supporting Information). Furthermore, all these cells, including microglia, showed a decrease in GAPDH expression (Figure S4, Supporting Information). This reduction in immune cell infiltration may be due to a requirement of glycolysis for immune cell migration across endothelial barriers.^[^ ^35^ ^]^ Alternatively, this might result from reduced MC numbers in the brain or an altered metabolic profile of microglia (Figure S4, Supporting Information), as both these cells contribute to the accumulation of other immune cells in the brain.^[^ ^24^, ^36^ ^]^ Irrespective, these findings collectively highlight the potential of glycolysis inhibition as a therapeutic approach to attenuate immune cell infiltration during severe CNS inflammation.

### 2.5 Glycolysis Inhibition Reduces Monocyte Infiltration into the CNS, but does not Reduce BM Myelopoiesis

**Figure 5:**
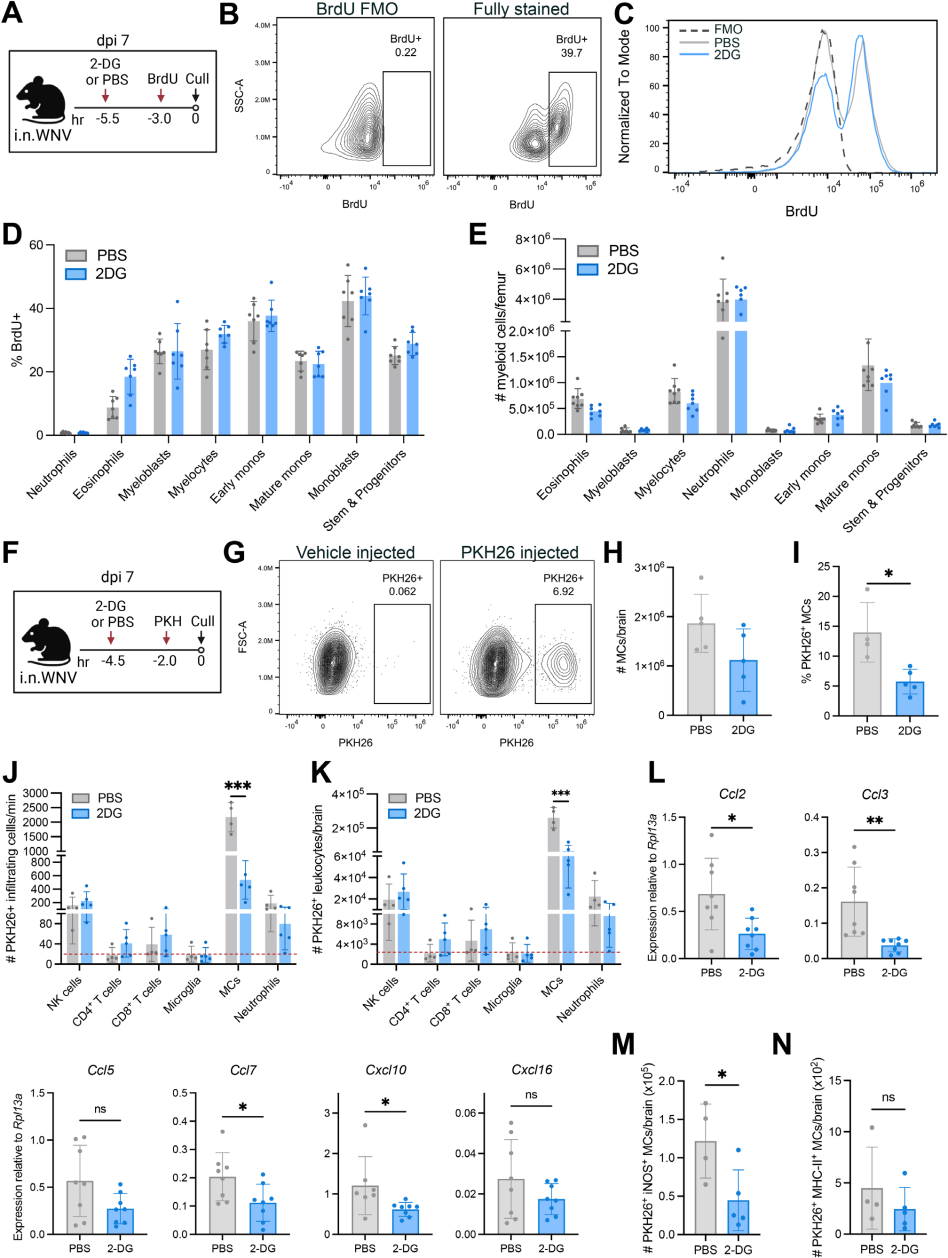
Glycolytic inhibition with 2‐DG reduces CNS infiltration without impacting BM myelopoiesis. A) Schematic showing treatment regimen. Mice infected with WNV received 2‐DG or PBS treatment once daily from 4–6 dpi and 5.5 h prior to euthanasia at 7 dpi. BrdU was administered to both groups 2.5 h after 2‐DG treatment at 7 dpi to assess differential rates of cellular proliferation. B) Contour plots showing BrdU incorporation in BM‐derived monocytes compared to a fluorescence minus one (FMO) control. C) Histogram depicting BrdU expression in mature monocytes from 2‐DG‐treated versus PBS‐treated mice. D) Proportion of BM myeloid cells incorporating BrdU. E) Absolute numbers of indicated myeloid cell subsets in the femur. F) Schematic showing the experimental workflow for tracking CNS infiltration. Mice were treated with 2‐DG once 4.5 h before euthanasia at 7 dpi, followed by an injection with PKH26 2.5 h later for in vivo labelling of blood and BM cells. G) Contour Plot showing PKH26 staining in brain MCs compared to the FMO control (i.e., vehicle‐injected mice). H) Total number of MCs present in the brain at 7 dpi. I) Percentage of PKH26^+^ MCs in the brain (i.e., recently infiltrating cells). J,K) Absolute numbers of indicated CD45^+^, PKH26^+^ leukocyte subsets infiltrating the brain per minute (J) or total accumulated cells in the brain (K). L). mRNA expression for selected chemokines in the brains of PBS and 2‐DG‐treated mice at dpi 7, as determined by qPCR. Expression was normalized to the housekeeping gene *Rpl13a*. Mice were treated once daily as depicted in (A). M,N) Absolute numbers of PKH26^+^ iNOS^+^ MCs (M), and PKH26^+^ MHC‐II^+^ MCs (N) within the brain. Data is representative of one independent experiment with 4–8 mice per group. Dotted red line in J, K is indicative of threshold for background PKH26^+^ staining as determined by positive staining of microglia. Statistics calculated by unpaired *t*‐test (H, I, M, N) or multiple t‐tests with two‐stage step up method of Bejamini, Krieger, and Yekutieli tests for multiple comparisons (D, E, J, K). * *p*<0.05, *** *p*<0.001. Error bars are representative of mean ± SD.

Glycolysis has been shown to be important for both myelopoiesis and cellular migration. We have previously shown that diminished myelopoiesis correlates with reduced brain MC numbers and better clinical outcomes in WNV infection.^[^ ^32^ ^]^ Thus, to investigate whether the protective effect of 2‐DG was due to a reduction in myelopoiesis and/or subsequent CNS infiltration, we first measured changes in monocyte proliferation using BrdU, which incorporates detectably into synthesising DNA (**Figure** **5**A,B). Our data revealed no significant changes in BrdU incorporation following 2‐DG treatment (Figure 5C,D), with proliferating cell proportions consistent across all monocyte differentiation phases and other myeloid cell types (Figure 5D; Figure S5, Supporting Information). Correspondingly, total BM monocyte counts were comparable between PBS‐ and 2‐DG‐treated mice (Figure 5E).

We then assessed the effect of 2‐DG on cellular migration into the brain. WNV‐infected mice were administered a single dose of 2‐DG at 7 dpi followed by intravenous injection of the fluorescent dye, PKH26 (Figure 5F,G). This labels blood and BM cells in the vasculature in vivo (Figure 5F,G), thereby enabling the discrete identification of cells that have infiltrated into the brain from the periphery after PKH26 injection.^[^ ^32^ ^]^ Post‐treatment analysis showed no significant change in total brain MC numbers, compared to PBS‐treated, WNV‐infected mice (Figure 5H). However, there was a significant decrease in the proportion of PKH26^+^ MCs (Figure 5I) and a marked reduction in the infiltration rate of PKH26^+^ MCs into the brain, but no reduction in the rate of infiltration of other dye‐positive leukocytes (Figure 5J). This corresponded to a significant decrease in the total number of PKH26^+^ MCs in the brain not evident in other infiltrating cells (Figure 5K). This indicates an acute MC‐specific effect of 2‐DG on MC migration into the brain. It also strongly suggests that the reduced presence of other immigrating leukocytes after longer term treatment with 2‐DG from 4–7 dpi (Figure 4I,J) is a consequence of reduced recruitment occasioned by the accumulation of fewer MCs in the brain, rather than the dependence on glycolysis per se for diapedesis by non‐MCs. Indeed, daily 2‐DG treatment significantly reduced the expression of chemokines recruiting various immune cells to the brain, including *Ccl2*, *Ccl7*, *Ccl3*, and *Cxcl10* (Figure 5L). *Cxcl16*, produced by astrocytes and microglia, was not reduced, suggesting that 2‐DG treatment does not affect chemokine production by brain‐resident cells. Furthermore, 2‐DG treatment did not affect the absolute numbers of effector and memory T cells, their proliferation, nor their GAPDH levels in the cervical lymph nodes draining the brain following daily 2‐DG treatment from 4–7 dpi (Figure S6, Supporting Information), indicating that the reduced T cell numbers in the bain is not due to systemic effects of 2‐DG on T cell expansion in the lymph nodes. Taken together, this is consistent with findings that inhibiting monocyte brain accumulation, e.g., via Ly6C blockade or clodronate liposome administration results in significantly reduced T cell and NK cell infiltration.^[^ ^24^, ^36^ ^]^ Additionally, we found that 2‐DG preferentially reduced the number of dye‐positive iNOS^+^ MCs compared to MHC‐II^+^ MCs in the brain (Figure 5M,N), suggesting that glycolysis inhibition may preferentially impede the differentiation of iNOS^+^ MCs once in the brain, likely due to their particular reliance on glycolysis.

### 2.6 Glycolysis Inhibition Differentially Affects NO‐Producing and Antigen‐Presenting Capacity of Myeloid Cells

**Figure 6:**
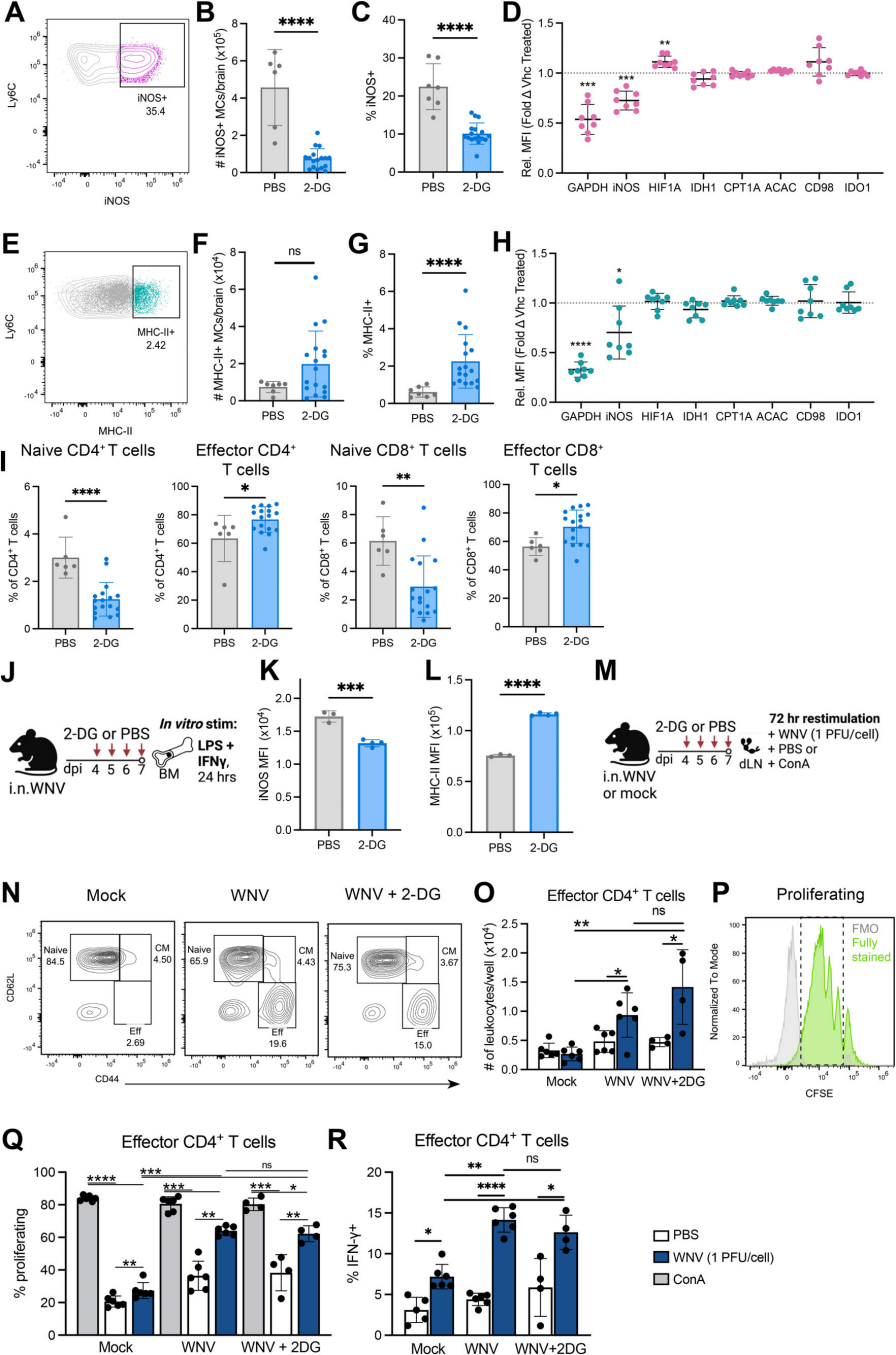
Glycolysis inhibition differentially affects iNOS^+^ and MHC‐II^+^ MCs. A) Dot plot showing iNOS^+^ MCs in the brain at 7 dpi. B,C) Number (B) and percent (C) of iNOS^+^ MCs in the brain at 7 dpi. D) Fold‐change in MFI of selected metabolic markers in 2‐DG‐treated mice relative to PBS‐treated mice in iNOS^+^ MCs. E) Dot plot showing MHC‐II^+^ MCs in the brain at 7 dpi. F,G) Number (F) and percent (G) of MHC‐II^+^ MCs in the brain at 7 dpi. H) Fold‐change in median fluorescence intensity of selected metabolic markers in 2‐DG‐treated mice relative to PBS‐treated mice in MHC‐II^+^ MCs. I) Percent of naïve and effector CD4^+^ and CD8^+^ T cells in the brains of 2‐DG‐treated and PBS‐treated mice. J) Schematic outlining experimental design for BM stimulation. Whole BM cells were isolated from WNV‐infected mice treated with 2‐DG or PBS daily from day 4–7, and then stimulated with LPS and IFN‐γ for 24 h. K,L) MFI of iNOS (K) and MHC‐II (L) in cultured monocytes. M) Schematic showing experimental design for assessment of T cell proliferation. Whole cervical draining lymph nodes (dLN) were stimulated with WNV or PBS for 1 h, prior to being cultured with either PBS or Con A for 72 h. N) Contour plots showing gating of naïve, central memory (CM) and effector (eff) CD4^+^ T cells based on CD62L and CD44 expression across groups stimulated with WNV. O) Number of effector CD4^+^ T cells per well of cultured lymph nodes. P) Histogram depicting CFSE staining relative to fluorescence‐minus‐one control (FMO). The dotted box indicates proliferating cells. Q,R) Percent of proliferating (Q) and IFN‐γ^+^ (R) effector CD4^+^ T cells. Data is from one independent experiment with eight mice per group. Statistics calculated ordinary one‐way ANOVA with Tukey's test for multiple comparisons (D, H), Mann‐Whitney test (B, C, F, G, I, K, L), RM one‐way ANOVA with Tukey's test for multiple comparisons (O, Q, R) and paired *t*‐test (N, Q). * *p* < 0.05, ** *p* < 0.01, *** *p* < 0.001, **** *p* < 0.0001. Error bars are representative of mean ± SD.

We next aimed to determine whether systemic glycolysis inhibition differentially affects MC subsets. To do this, all iNOS^+^ and MHC‐II^+^ MCs were manually gated for analysis (**Figure** **6**A,E; Figure S7, Supporting Information). To determine the extent of overlap between inflammatory (iNOS⁺) and antigen‐presenting (MHC‐II⁺) MC subsets via manual gating, we quantified the proportion of each population that co‐expressed the other marker. Less than 10% of MHC‐II⁺ MCs were iNOS⁺, and fewer than 1% of iNOS⁺ MCs expressed MHC‐II, indicating that these subsets are largely distinct with minimal phenotypic overlap (Figure S7, Supporting Information).

Metabolic profiling revealed an >80% decrease in numbers of iNOS^+^ MCs within the inflamed brain following daily 2‐DG treatment for five days (Figure 6B), mirroring a similar decline in the proportion of MCs expressing iNOS (Figure 6C). Importantly, 2‐DG treatment only affected the expression of markers related to glycolysis (GAPDH) and NO production (iNOS) in MCs (Figure 6D), demonstrating that 2‐DG treatment selectively reduced glycolysis without impacting other metabolic pathways. This was borne out by a significant increase in HIF1‐α with 2‐DG treatment (Figure 6D). This pattern emphasizes the specificity of 2‐DG on glycolysis, with negligible effects on other examined metabolic processes. Furthermore, clustering 2‐DG‐treated and PBS‐treated BM and brain myeloid cells on metabolic proteins revealed no significant suppression of the HIF1‐α^+^ intermediate population (cluster 2) in the brain at dpi 7 (Figure S8, Supporting Information). This observation supports the hypothesis that this intermediate subset, in contrast to the iNOS^+^ MC population, has a reduced reliance on glycolytic processes.

Despite the substantial decrease in iNOS⁺ MCs, the number of MHC‐II⁺ MCs in the brain was not reduced by 2‐DG treatment (Figure 6F), resulting in a three and fourfold proportional increase (Figure 6G), respectively. In these cells, 2‐DG caused only minor reductions in GAPDH and iNOS expression (Figure 6H), consistent with their already lower glycolytic profile. Importantly, the proportional increase in MHC‐II^+^ MCs was accompanied by a marked reduction in the proportion of naïve CD4⁺ and CD8⁺ T cells in the brain, and a corresponding increase in effector T cell proportions of both lineages with 2‐DG treatment (Figure 6I). This phenotypic transition supports the notion of enhanced T cell activation and provides complementary evidence suggesting that the increased proportion of MHC‐II⁺ APC‐MCs may contribute to ongoing antigen presentation and expansion of effector T cells during treatment.

To examine the functional implications of glycolysis inhibition in more detail, BM cells isolated from 2‐DG‐ and PBS‐treated WNV‐infected mice were stimulated with classical M1 activation stimuli (IFN‐γ + LPS) in vitro (Figure 6J). Monocytes from 2‐DG‐treated mice exhibited significantly reduced expression of iNOS relative to vehicle‐treated WNV‐infected mice (Figure 6K), suggesting that BM monocytes from 2‐DG‐treated mice have reduced capacity for NO production in response to inflammatory stimuli. Interestingly, although these cells significantly reduced iNOS expression, BM monocytes from 2‐DG‐treated mice showed significantly increased MHC‐II expression in response to inflammatory stimulation relative to control‐treated mice, presumably due to IFN‐γ exposure (Figure 6L). This further suggests that while systemic 2‐DG treatment may reduce the inflammatory potential of monocytes prior to their differentiation into effector MCs, it may not affect, or may even improve, their capacity for antigen presentation.

To confirm that antigen‐presenting functions are not impacted by 2‐DG, we isolated draining cervical lymph nodes from 4–7 dpi 2‐DG‐ and vehicle‐treated WNV‐infected mice and vehicle‐treated mock‐infected mice at 7 dpi and stimulated them with WNV isolates, which contains both replicating virus and free viral antigen, for 72 h (Figure 6M). Effector CD4^+^ T cell differentiation (Figure 6N) was significantly increased following in vitro viral restimulation of T cells isolated from WNV‐infected mice treated with vehicle or 2‐DG, compared to those from mock‐infected mice (Figure 6O). Importantly, 2‐DG‐treated and vehicle‐treated WNV‐infected mice showed comparable numbers of effector CD4^+^ T cells, suggesting that the capacity of antigen‐presenting cells to stimulate an effector T cell response is unaffected by 2‐DG treatment. Supporting this, the proportions of proliferating effector CD4^+^ T cells (Figure 6P,Q) and IFN‐γ‐producing effector T cells (Figure 6R) in response to viral re‐stimulation were also unaffected by 2‐DG treatment. CD8^+^ T cells remained similarly unaffected by 2‐DG treatment (Figure S9, Supporting Information). Together, these findings demonstrate that glycolysis inhibition selectively targets inflammatory, NO‐producing MCs without compromising antigen presentation or downstream T cell responses.

## 3 Conclusion

This study represents an integrated approach using both scRNA‐seq and spectral cytometry to inspect the metabolic profiles of MCs at a gene and protein level, uncovering for the first time the intricate metabolic diversity of these cells within the context of WNV encephalitis. The novelty of this study lies in the observation that MCs, arising from three metabolically distinct subsets in the BM, transit to the brain and further differentiate into three distinct subsets. The first of these, a HIF1‐α^+^ population, gives rise to two further metabolically and functionally distinct populations: a glycolytic NO‐producing population, clearly contributing to severe neuroinflammation, and a second, non‐glycolytic population with antigen‐presenting capabilities, potentially supporting ongoing T cell responses important for viral clearance. This suggests a continuous regulatory capacity in MC metabolism that controls functional differentiation. Targeting the NO‐producing population with the glycolysis inhibitor, 2‐DG, specifically reduced their migration into the brain and impaired their ability to produce NO. Strikingly, this corresponded with a significant reduction in clinical and neuroinflammatory signs, highlighting the therapeutic potential of modulating immunometabolism to ameliorate disease.

Our findings indicate that myeloid cells adopt multiple functional roles in the infected brain, each defined by a unique metabolic phenotype. The local tissue environment was likely a decisive factor influencing these metabolic patterns, as BM monocytes and brain MCs demonstrated distinct metabolic signatures. This observation is consonant with recent research highlighting the significance of tissue origin in shaping the metabolic characteristics of resident macrophages,^[^ ^8^ ^]^ in which the varying nutritional and cellular contexts provided by different tissues likely drive cells toward specific metabolic pathways. Furthermore, the divergent developmental origins of microglia (arising from the yolk sac) and MCs (originating from hematopoietic stem cells in the BM) likely contributed to metabolic differences during infection. Notably, microglia tended to maintain a more homeostatic metabolic state, while MCs shifted toward more active metabolic profiles. This could reflect a protective function of resident microglia, maintaining a more robust and regulated state to prevent excessive damage, thereby safeguarding non‐regenerating neurons and limiting neurodegeneration. Overall, this distinction emphasizes the importance of both origin and environment in the metabolic identity of immune cells in the CNS during infection.

The metabolic programming of monocytes—whether established during development in the BM or upon entry into the virus‐infected CNS—remains unclear. Our findings suggest metabolic cues in each organ play an important role, with trajectory analysis revealing a clear metabolic progression of monocytes migrating from the BM to the CNS. This transition is marked by a decreased expression of genes involved in ATP production, the TCA cycle, and the ETC, coupled with an increased reliance on glycolysis. While the developmental trajectory of metabolic clusters in the BM remains to be fully elucidated, our observations indicate that cluster 1, initially expanding in the early stages of infection but diminishing by dpi 7, may evolve into more metabolically active clusters 2 and 3. This initial metabolic state of BM cells could set the stage for varied differentiation pathways of MCs, effectively “priming” them before they migrate to the inflamed brain environment. In addition, although spatial compartmentalization within the CNS has been proposed to influence MC differentiation,^[^ ^37^, ^38^, ^39^ ^]^ our data suggest that the divergence between the identified MC subsets in the brain is primarily temporal. Antigen‐presenting MCs (cluster 2) peak at 5 dpi, whereas iNOS⁺ MCs (cluster 7) emerge predominantly at 7 dpi, suggesting that evolving microenvironmental cues during disease progression may drive their emergence. This highlights the dynamic adaptation of MCs to the CNS inflammatory milieu and suggests a defined therapeutic window during which their metabolic state—and potentially their function—may be selectively modulated. Nonetheless, we cannot exclude the possibility that these subsets also represent spatially distinct populations. Future studies are needed to determine whether they arise from unique anatomical compartments such as perivascular or choroid plexus‐associated niches, particularly given the inability to distinguish infiltrating MCs from border‐associated macrophages in our current approach. We acknowledge this as a limitation and a key avenue for future investigation.

Once in the brain, BM monocytes evidently assume a HIF1‐α^+^ intermediate state, which may act as the branching point for divergence into iNOS^+^ or APC MC subsets. Such a metabolic shift might be an adaptive strategy to ensure survival in the hypoxic conditions of the infected CNS.^[^ ^40^ ^]^ Importantly, HIF1‐α was significantly upregulated in glycolytic MCs in our study and maintained its expression in infiltrating MCs following 2‐DG treatment, indicating its independence from glycolysis. This presumably enables immigration of APC MCs, but not iNOS^+^ MCs. Several factors could explain this observed independence between HIF1‐α signalling and glycolysis. While HIF1‐α is widely recognized as a regulator of glycolysis, its expression can also be sustained by alternative stimuli such as inflammatory signalling or reactive oxygen species,^[^ ^41^, ^42^ ^]^ and it may serve context‐specific roles beyond metabolic control. In myeloid cells, HIF1‐α has been shown to promote migration and survival under inflammatory conditions,^[^ ^17^, ^18^ ^]^ supporting a potential role in guiding MC trafficking rather than directly controlling glycolytic flux. Additionally, other pathways, including PI3K/AKT and c‐Myc signalling, can independently drive glycolytic gene expression,^[^ ^43^ ^]^ potentially decoupling HIF1‐α from metabolic regulation in this context. Future studies employing cell‐specific conditional knockouts of HIF1‐α and glycolytic enzymes will be critical to dissect the relative contributions of these regulatory mechanisms.

Additionally, 2‐DG treatment significantly reduced physical monocyte migration into the inflamed brain without impacting myelopoiesis, suggesting that glycolytic inhibition mediates its protective effect by preferentially preventing inflammatory cellular transmigration across the blood‐brain barrier. The cellular specificity of this targeting may be due to the entry of these inflammatory cells at distinct anatomical locations in the CNS or their exposure to different regions of the brain that have different rates of infection and unique cytokine or chemokine profiles.^[^ ^44^ ^]^ This link is substantiated by observations in experimental autoimmune encephalomyelitis and human multiple sclerosis, where heightened glycolytic activity is associated with the transmigration of inflammatory macrophages into the CNS.^[^ ^45^ ^]^ However, as 2‐DG treatment reduced chemokine expression in the brain, it remains unclear whether the differential effect of 2‐DG on the migration of APC and iNOS^+^ MCs stems from inherent differences in migratory capacity or their dependence on different chemokine gradients. Moreover, determining whether the observed decrease in chemokine levels is a consequence of the attenuated migration of MCs into the brain is challenging. This is of particular importance since MCs are essential for the synthesis of chemokines that recruit immune cells to the WNV‐infected brain.^[^ ^24^ ^]^

Enhanced glycolysis in immune cells during WNV encephalitis and other inflammatory diseases suggests that targeting metabolic reprogramming within inflammatory monocytes could be a promising strategy to modulate disease severity. In this study, 2‐DG treatment selectively impacted pathogenic, NO‐producing MCs, while sparing antigen‐presenting subsets. The ability of 2‐DG to cross the blood‐brain barrier^[^ ^46^ ^]^ likely facilitates direct modulation of these cells within the CNS. Supporting this, we observed a reduction in inflammatory gene and cytokine expression in the brain following 2‐DG treatment, consistent with previous findings^[^ ^47^ ^]^ and indicative of a broader anti‐inflammatory effect. Although 2‐DG may also affect glycolysis in non‐immune cells such as neurons,^[^ ^47^ ^]^ several lines of evidence support a direct effect on monocytes. First, CNS viral burden was unchanged by treatment, arguing against impaired viral replication in neurons. Second, previous work in this model has established that NO‐producing MCs, not neurons, are the primary drivers of inflammation and pathology.^[^ ^21^, ^22^, ^23^, ^48^ ^]^ Third, we observed selective suppression of iNOS⁺ MCs while antigen‐presenting cells were maintained, suggesting cell‐type–specific vulnerability rather than indirect neuronal modulation. This aligns with a model in which glycolytic inhibition preferentially affects highly metabolically active monocyte subsets. Indeed, similar selectivity has been demonstrated in experimental autoimmune encephalomyelitis, where 2‐DG treatment skewed monocytes/macrophages toward an anti‐inflammatory state and improved clinical outcomes.^[^ ^46^ ^]^

Interestingly, we show that antigen‐presenting MCs have a metabolic profile distinct from those producing NO, primarily attributed to reduced glycolytic activity. Although we did not employ Seahorse flux assays or metabolomics — which require high cell numbers not attainable for rare subsets such as MHC‐II⁺ MCs — we used an integrated approach combining transcriptomic profiling, metabolic protein analysis via MetFlow, and functional glycolysis inhibition with 2‐DG. This strategy allowed us to examine metabolic differences at both the gene and protein level and to test the functional importance of glycolysis in shaping MC phenotypes. Notably, MetFlow has been shown to correlate well with Seahorse‐derived glycolytic activity in macrophages,^[^ ^8^, ^29^ ^]^ supporting its utility as a surrogate platform in settings where cell number is limiting. Consistent with our findings, MHC‐II^hi^ macrophages also displayed lower reliance on glycolysis than MHC‐II^lo^ macrophages,^[^ ^49^ ^]^ and monocytes diminish glycolytic activity as they develop into an antigen‐presenting phenotype in vitro,^[^ ^50^ ^]^ suggesting a negative association between glycolysis and antigen presentation. Supporting this, elevated glucose levels have been shown to hinder antigen presentation and disrupt CD4^+^ T cell activation.^[^ ^51^ ^]^ Conversely, glucose limitation appears to enhance MC‐mediated T cell responses, as demonstrated by the increased expression by glucose‐deprived MCs of co‐stimulatory molecules and interleukin‐12 essential for T cell proliferation and function.^[^ ^52^ ^]^ Thus, APC may strategically reduce glycolysis to adapt to the glucose‐scarce environment generated by metabolically‐active T cells, thereby prolonging the T cell response.^[^ ^52^ ^]^ In this study, MCs may transition to an antigen‐presenting role as their glycolytic activity declines to optimize their capacity to present antigens effectively. This study did not evaluate the antigen‐presenting capability of MCs from the brain due to limited cell numbers and the challenges associated with primary brain cell culture. However, APCs isolated from the draining lymph nodes of 2‐DG treated mice maintained their ability to elicit an anti‐viral T cell response after antigen rechallenge *ex vivo*. These findings indicate that 2‐DG does not diminish the antigen presenting efficacy of APCs and does not impair the establishment of a memory T cell response.

The unaffected memory T cell response during glycolytic inhibition may be attributed to their reliance on fatty acid oxidation, a metabolic pathway essential for memory development.^[^ ^53^, ^54^ ^]^ Importantly, while 2‐DG treatment significantly supressed glycolytic markers, it did not alter other metabolic pathways, including fatty acid oxidation. Consequently, despite a reduction in T cell numbers in the brain over three days of 2‐DG treatment, T cell proliferation and memory formation evidently remained intact. Furthermore, the absence of acute inhibition of T cell immigration by 2‐DG, in contrast to iNOS^+^ MCs, strongly suggests that reduced T cell infiltration into the brain by longer term 2‐DG treatment was more likely a consequence of their reduced recruitment by low MC numbers than direct migration inhibition of these cells by 2‐DG. In summary, our findings suggest that glycolytic inhibition selectively hinders the infiltration of hyperinflammatory cells without affecting the functional development of a robust T cell response during WNV infection.

This research highlights that modulating the metabolic pathways active in pathogenic monocytes can mitigate disease severity by specifically tempering uncontrolled inflammation, a key contributor to disease exacerbation and progression. This nuanced approach preserves essential immune processes, including pathogen clearance and memory formation, and may be most effective when combined with anti‐viral therapies. Unlike broad‐acting immunosuppressants like corticosteroids, which indiscriminately suppress both detrimental and beneficial immune responses, metabolic modulation offers more targeted control of the inflammatory response. Although the effectiveness of this strategy in humans requires further study, these findings reinforce the potential of metabolic targeting to complement, e.g., anti‐microbial treatment, as a component of combination therapy for immune regulation in diseases with severe or uncontrolled inflammation.

## 4 Experimental Section

### Mice

Female 9 to 10‐week‐old C57BL/6 mice were obtained from Animal BioResources (NSW, Australia) or the Animal Resource Centre (WA, Australia). All experiments involving mice were approved by the University of Sydney Animal Ethics Committee (Protocol 1696). Mice maintained specific pathogen‐free at the Charles Perkins Centre (Sydney, Australia) under Biosafety Level (BSL) II conditions. Mice were group‐housed in individually ventilated cages at ≈21 °C and a relative humidity of 45–46% on a 12 h day per night light cycle with ad libitum access to standard rodent chow and water. Sterile cardboard and shelter were provided for environmental enrichment. Mice were euthanised by intraperitoneal injection of 200 µL of Avertin, followed by transcardial perfusion with 10 mL of phosphate‐buffered saline (PBS) and subsequent decapitation. It was elected to use female mice as animals are required to be housed together for ethical and scientific purposes. Inflammatory injuries associated with housing dominant males in groups produces background immunological reactions which may confound results. There is no evidence to suggest that the immunological response to WNV encephalitis differs between males and females.

### Study Design and Data Inclusion

For all treatment groups, mice were randomised to experimental groups at the cage level, with each cage assigned to a specific treatment or control group to minimise potential cage‐related confounders. Order of treatments was randomised to minimise potential confounders. Procedures were not performed until after a week of acclimatisation. The investigator conducting the study was aware of group allocations during all stages of the primary experiments, including group assignment, experimental procedures, outcome assessment, and data analysis.

Mice were included in the analysis if they showed evidence of successful infection, defined by the presence of ≥1 × 10⁶ total cells in the brain and/or detectable virus in brain homogenates as determined by plaque assay at 7 days post‐infection (dpi). Mice that did not meet these criteria were excluded. For statistical analyses, outliers were assessed using the ROUT method (Q = 1%); however, no samples were excluded on this basis. All inclusion and exclusion criteria were pre‐established and applied consistently across experiments.

### WNV Infection

For WNV infection, mice were anesthetized with isoflurane and infected intranasally with a LD100 dose (3 × 10^4^ plaque‐forming units) of WNV (Sarafend), as described previously.^[^ ^48^ ^]^ Mice were culled at the indicated timepoints in each experiment.

### 2‐deoxy‐d‐glucose (2‐DG) Treatment

2‐DG (Sigma‐Aldrich, USA) was injected intraperitoneally once daily from 4–7 days post‐infection (dpi) at a dose of 2 g kg^−1^ body weight in 200 µL of sterile PBS. PBS was used as a vehicle control. In some experiments, 2‐DG (2 g kg^−1^) was administered once at 7 dpi at either 4.5 h or 5.5 h before euthanasia.

### Detection of Proliferating Cells with BrdU

Bromodeoxyuridine (BrdU) (Sigma‐Aldrich, USA) was injected intraperitoneally 3 h before sacrifice at a dose of 1 mg prepared in 200 µL of sterile PBS.

### Detection of Nitric Oxide

NO was detected by using DAF‐FM diacetate (Invitrogen, USA), which is a cell‐permeable fluorescent probe that is nonfluorescent until it reacts with NO. Cells were resuspended in DAF‐FM (5 nmol mL^−1^) in PBS for 30 min at room temperature, prior to cell surface staining. DAF‐FM staining was detected flow cytometrically.

### Tracking Recently Infiltrating Cells into the CNS with PKH26

PKH26 cell linker was combined with diluent C (Sigma‐Aldrich, USA) at a tenfold higher concentration than the manufacturer's recommendation and injected intravenously via the lateral tail vein 2 h before euthanasia, as previously described.^[^ ^32^ ^]^

### Quantification of Viral Titre Using a Plaque Assay

Baby Hamster Kidney (BHK) fibroblasts were used to perform a plaque assay, as previously described.^[^ ^48^ ^]^ Briefly, BHK cells were inoculated with brain tissue homogenates in a series of tenfold dilutions for 1 hr, then subsequently overlaid with agarose. After a 3‐day incubation, cells were fixed with 10% formalin (Sigma‐Aldrich, USA), then stained with 1% crystal violet solution. Viral load, expressed as plaque‐forming units (PFU) per gram of brain tissue, was calculated by quantifying the number of visible plaques and accounting for inoculum volume and dilutions used.

### RNA Extraction and Real‐Time Quantitative Polymerase Chain Reaction

Brain samples were homogenized using TRI Reagent (Sigma Aldrich, USA) and a tissue homogenizer (TissueLyzer, Qiagen, DE). The High‐Capacity cDNA Reverse Transcription Kit (ThermoFisher Scientific, USA) was used to synthesize cDNA and The Power SYBR Green PCR Master Mix (ThermoFisher Scientific, USA) was then used for real‐time quantitative polymerase chain reaction (qPCR) using primers detailed in Table S2 (Supporting Information). This was conducted using the LightCycle 480 Instrument II (Roche, CH). Gene expression values were normalized to *Rpl13a*.

### Tissue Processing

The brain, BM, and cervical lymph nodes (LN) were collected from mice anesthetized with an intraperitoneal injection of avertin and perfused transcardially with PBS. Cells from the BM were isolated by flushing the femur with PBS using a 30‐gauge needle. Red blood cells were then lysed using 1x Pharm Lyse Buffer (BD Biosciences, USA). Brains were digested enzymatically with DNase I (0.1 mg mL^−1^, DN25, Sigma‐Aldrich, USA) and collagenase type IV (1 mg mL^−1^, C5138, Sigma‐Aldrich, USA) using the gentleMACS dissociator (Miltenyi Biotec, DE). A 30/80% Percoll gradient was then used to isolate leukocytes from brain homogenates. Cervical lymph nodes were gently mashed through a 70 µm nylon mesh sieve using a syringe plunger. Live cells were counted with trypan blue (0.4%) on a hemocytometer.

### Cell Culture—T Cell Proliferation Assay

Five million LN cells were stained with 1.5 µM of Carboxyfluorescein succinimidyl ester (CFSE) (Life Technologies) in 1 mL of PBS, as previously described.^[^ ^55^ ^]^ CFSE^+‐^cells (5 × 10^5^ cells/well with two technical replicates) were then cultured for 1 hr at 37 °C and 5% CO^2^ in media or 50 µL of WNV solution (1 PFU/cell) with agitation every 10 min. Media was composed of RPMI (Lonza Biosciences, USA) supplemented with 1 U of HEPES (Sigma‐Aldrich, USA), 5% FCS, 0.1 M of b‐mercaptoethanol (Gibco), and 1x penicillin streptomycin (Thermofisher Scientific). Unbound virus was then washed off and cells were cultured for a further 72 h in media alone or media supplemented with 1 ug mL^−1^ of Concanavalin A (Sigma), as a positive control for T cell proliferation.

### BM Stimulation

BM cells (1.2 × 10^6^ cells per well with two technical replicates) were stimulated with 100 ng mL^−1^ IFN‐γ (Biolegend, USA) for 4 h and 100 ng mL^−1^ of lipopolysaccharide (LPS) (InvivoGen) for a total of 24 h in a non‐adherent 96‐well plate (Corning).

### Spectral Flow Cytometry

Single‐cell suspensions were stained with anti‐CD16/32 (fluorescently conjugated or unconjugated, Biolegend, USA) and Zombie UV Fixable Viability kit (Biologend, USA) for 30 min on ice, washed twice, and subsequently stained with MitoSOX in PBS for 30 min at 37 °C. Cells were then washed twice and stained with a cocktail of fluorescently‐labelled surface‐stain antibodies in FACS on ice for 30 min. Cells were washed twice and permeabilized with True‐Nuclear 1x Fix Concentrate (Biolegend, USA) for 1 h at room temperature prior to staining with an intracellular/intranuclear antibody cocktail for 1 h on ice, washed twice, and then stained with secondary antibodies targeting ACAC and CPT1α for 1 hr on ice. Cells were stained with APC‐BrdU (BD Biosciences, USA) as previously described.^[^ ^56^ ^]^ Briefly, cells were incubated with Cytofix/Cytoperm (BD Biosciences, USA) after surface staining and fixation, then incubated with Cytoperm Permeabilization Buffer Plus (BD Biosciences, USA) and 1 mg mL^−1^ of DNase (Sigma‐Aldrich, USA; 30 units/sample, >400 units per mg of DNase), then stained with anti‐BrdU antibody for 30 mins at room temperature. The 5‐Laser Aurora spectral cytometer (Cytek Biosciences, USA) was used to measure fluorescently tagged antibodies. Unstained controls for each specific condition were used as reference controls for spectral unmixing in each experiment. Acquired data was analysed using FlowJo (v10.8, BD Biosciences, USA). Quality control measures such as time, single cells, non‐debris, and Live/Dead staining were applied to exclude debris, doublets, and dead cells. Antibody details are provided in Table S1 (Supporting Information).

### Analysis of Spectral Flow Cytometry Data

For bioinformatics analyses, the FCS files were compensated and gated in FlowJo (BD Biosciences, USA). Monocytes (for BM samples) or MCs and microglia (for brain samples) were then exported and downsampled to a total of 3 × 10^5^ cells (1.5 × 10^5^ per organ). FCS files were formatted with the CyCombine package^[^ ^57^ ^]^ and Arcsinh transformed (cofactor = 6000) before being converted into a Seurat object using a custom R script using Seurat (v4.0).^[^ ^58^ ^]^ Two independent experiments were then batch integrated using rPCA in Seurat using all markers as integration anchors. All subsequent analysis was performed on data as a Seurat object using the Seurat pipeline.^[^ ^58^ ^]^ Uniform manifold approximation and projection (UMAP) and clustering analysis was performed using only metabolic features.

For all other analyses, the FCS files were compensated and gated down to individual cell populations prior to exporting cell proportions and MFI in FlowJo (BD Biosciences, USA). Cell proportions and live cell counts were used to quantify cell numbers. MFIs from positive populations, as determined by the isotype or fluorescence minus one (FMO) control, were used to quantify protein expression of metabolic targets.

### Single‐Cell RNA‐Sequencing

Monocyte‐derived cells (MCs) were sorted from the brain at 5 and 7 days post‐infection (dpi) and identified based on established gating strategies.^[^ ^24^, ^31^ ^]^ Mature bone marrow monocytes were similarly isolated at 7 dpi.^[^ ^24^ ^]^ To prevent microglial contamination, microglia were separately sorted and barcoded using a validated gating approach,^[^ ^31^, ^32^ ^]^ and excluded them from downstream analyses (Figure S1, Supporting Information). To ensure ample cells for sorting, tissues from two animals were combined per sample. Single cell suspensions underwent CD16/32 blocking and Zombie UV viability staining (Biolegend, USA) before incubation with a cocktail of fluorescently‐conjugated surface stain antibodies.^[^ ^24^ ^]^ Sorting was performed on a 10‐laser Influx Cell Sorter (BD Bioscences, USA) using the FACS Diva Program (BD Biosciences, USA) followed by sample barcoding with a mouse multiplexing kit using anti‐CD45 (BD Biosciences, USA), as previously described.^[^ ^24^ ^]^ Cells were then pooled, stained for viability with Calcein AM and Draq7, and quantified using a BD Rhapsody Scanner. Quality control for cell sorting included metrics such as doublet rate and viability percentages.

Cell capture was performed according to manufacturer's instructions. Briefly, single‐cell transcriptomics was conducted using the BD Rhapsody Express System, and lysed cells were processed for reverse transcription and exonuclease I treatment. Libraries were created using microbead‐captured single cell transcriptomes as per the manufacturer's protocol using the BD Rhapsody cDNA Kit (BD Biosciences, USA) and the BD Rhapsody Targeted mRNA Amplification Kit (BD Biosciences, USA). The BD Rhapsody Immune Response Panel (Cat. # 633753), consisting of 397 genes, and an additional 67 custom genes were used.

For sequencing, libraries were quantified using a Qubit Fluorometer (Thermo Fisher Scientific) and KAPA Library Quantification Kit (Roche), adjusted to 2 nM, and pooled in a mRNA:sample tag ratio of 12.5:1. Sequencing was performed on an Illumina NextSeq1000.

### Analysis of Single‐Cell RNA‐Sequencing Data

SevenBridges (Seven Bridges Genomics Inc., USA) was used to process counts (ie., distribution‐based error correction molecules per cell) from single‐cell RNA‐seq data. Seurat (v4.0) was used for pre‐processing and removal of doublets/multiplets and cells with high mitochondrial reads (+3 median absolute deviations from the mean). After stringent quality control measures and filtering out contaminating cells, the final dataset contained 6405 cells (Figure S1, Supporting Information). Normalization and variance stabilization was then performed using sctransform() prior to UMAP and clustering.

The R package Slingshot (v1.8)^[^ ^59^ ^]^ was used to define computationally imputed pseudotime trajectories from brain and BM MCs/monocytes at 7 dpi.

For module scores, genes for all functional and metabolic pathways were downloaded from the Mouse Genome Informatics database.^[^ ^60^ ^]^ Genes associated with the negative regulation of the module of interest were manually filtered out prior to filtering genes available in the dataset (Table S3, Supporting Information). Module scores for a particular functional or metabolic module were derived by running the function AddModuleScore in Seurat, with 16 control genes and 24 bins.

### Statistical Analysis

Prior to each analysis, we evaluated data normality using the Shapiro‐Wilk test. Outliers were assessed using the ROUT method (Q = 1% For normally distributed data, parametric tests were applied (t‐test for two‐group comparisons or ANOVA with Tukey's test for multiple comparisons for multiple groups). When the normality assumption was violated, we implemented non‐parametric alternatives (Mann‐Whitney U test for two‐group comparisons or Kruskal‐Wallis test with Dunn's test for multiple comparisons for multiple groups). The selected statistical test for each analysis is listed in the corresponding figure legend. Data is presented as mean ± SD unless indicated otherwise and the sample size for each statistical analysis is indicated in the corresponding figure legend. Statistical analyses were carried out in GraphPad Prism (version 10.2.0 for Mac, GraphPad Software, Boston, Massachusetts USA, www.graphpad.com).

## Conflict of Interest

The authors have no conflicts of interest to disclose.

## Supporting information



Supporting Information

Supplemental Table 1

## Data Availability

The data that support the findings of this study are openly available in Gene Expression Omnibus at https://www.ncbi.nlm.nih.gov/geo/, reference number 297492.
