# Supplementary material for: Metabolic Programming Drives Protective and Inflammatory Monocyte Fates in Viral Encephalitis
Source: Adv Sci (Weinh). 2025 Jul 14;12(33):e05844. doi: 10.1002/advs.202505844 (PMC12412471; doi:10.1002/advs.202505844)
Supplement: Supplementary file 1 — Supporting Information [file ADVS-12-e05844-s002.pdf]

ADVANCED SCIENCE
Open Access

Supporting Information

for *Adv. Sci.*, DOI 10.1002/advs.202505844

Metabolic Programming Drives Protective and Inflammatory Monocyte Fates in Viral Encephalitis

*Claire L. Wishart, Alanna G. Spiteri, Jian Tan, Laurence Macia and Nicholas J. C. King**

Supporting Information

Metabolic programming drives protective and inflammatory monocyte fates in viral encephalitis

Claire L. Wishart, Alanna G. Spiteri, Jian Tan, Laurence Macia, Nicholas J.C. King

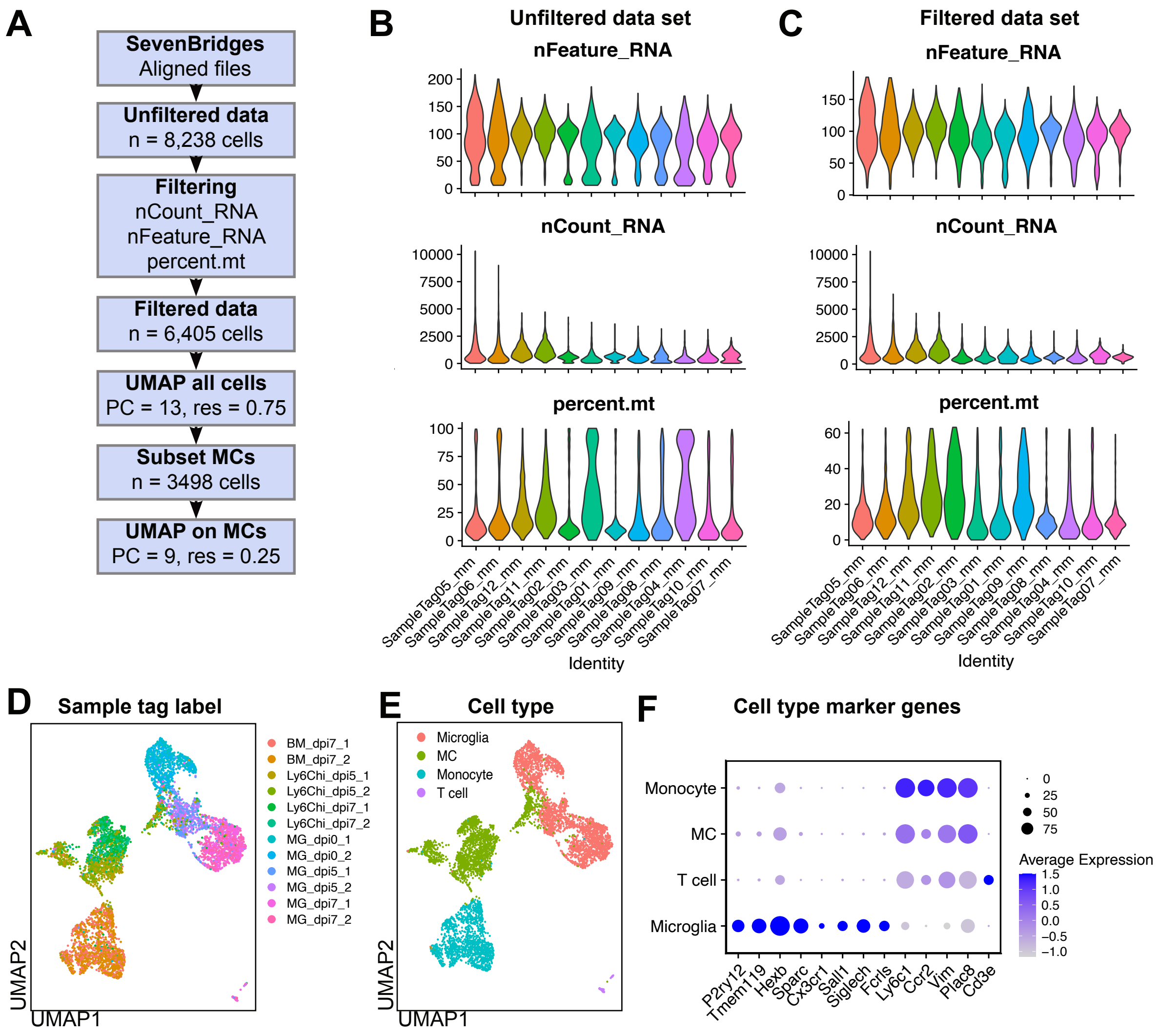

Figure S1. Quality control metrics and cell type classification for scRNA-seq data in the WNV infected brain and BM. (**A**) Analysis workflow. (**B**) Quality control metrics, including nFeature_RNA, nCount RNA, and percent mitochondrial gene expression before and after filtering for each of the sample tags (barcode). The unfiltered data set contained 8,238 cells and the filtered data set contained 6,305 cells. (**D**, **E**) UMAP showing clustering of filtered data set prior to subsetting MCs from microglia and T cells, pseudocoloured by sample tag (**D**) and cell type (**E**). (**F**) Dot plot heatmap showing the expression of select genes in identified cell types. MCs were subsetted from microglia according to sample tag label (**D**) and the expression of cell type-specific markers shown in (**F**). Data is from one experiment with two samples per group, with two mice pooled per sample (4 mice total per group).

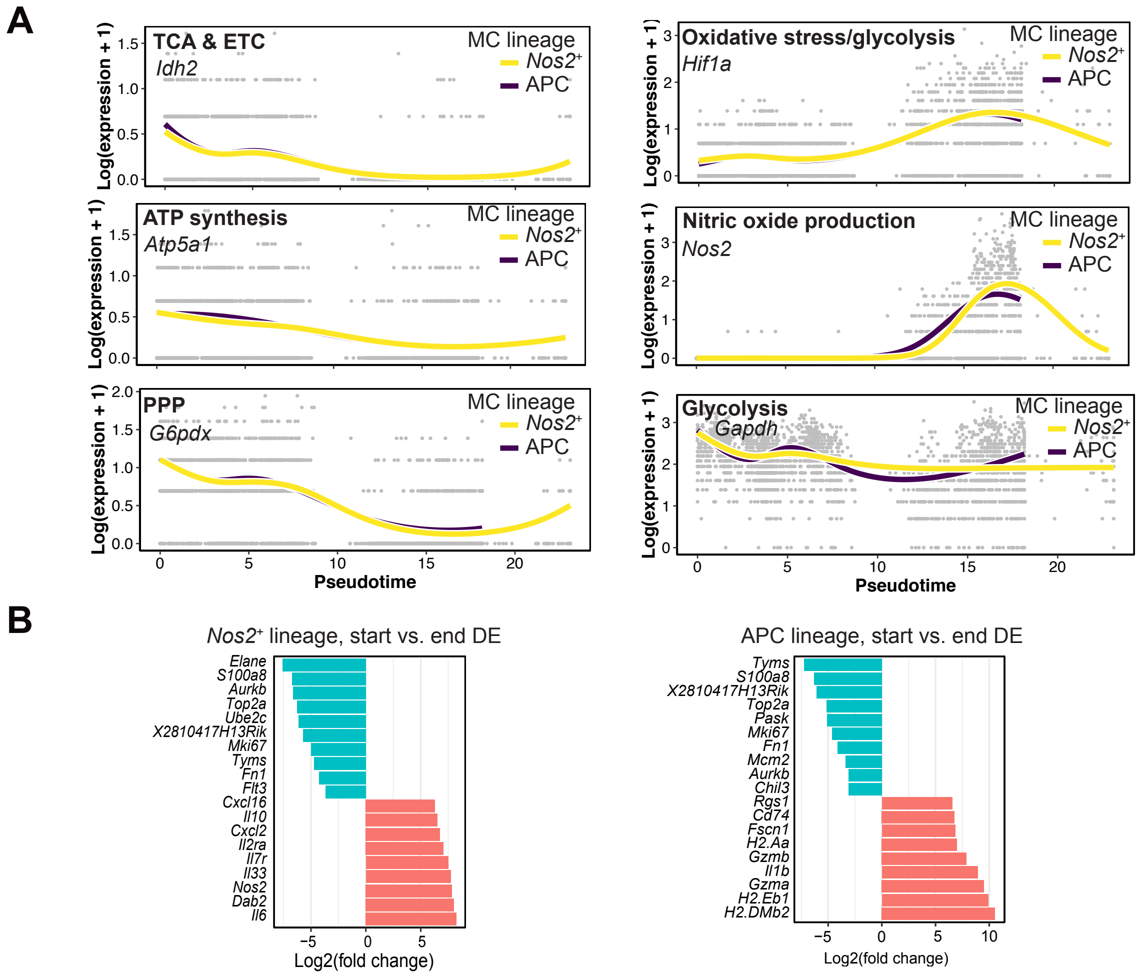

Figure S2. Pseudotemporal ordering of Nos2+ and APC lineages with Slingshot. (**A**) Expression of select genes in Nos2+ and APC lineages along pseudotime. (**B**) Top differentially expressed markers between the lineage root (*Mo3*) to lineage end-point (*Nos2+ MC* or *APC MC*) for each lineage. Data is from one experiment with two samples per group, with two mice pooled per sample (4 mice total per group).

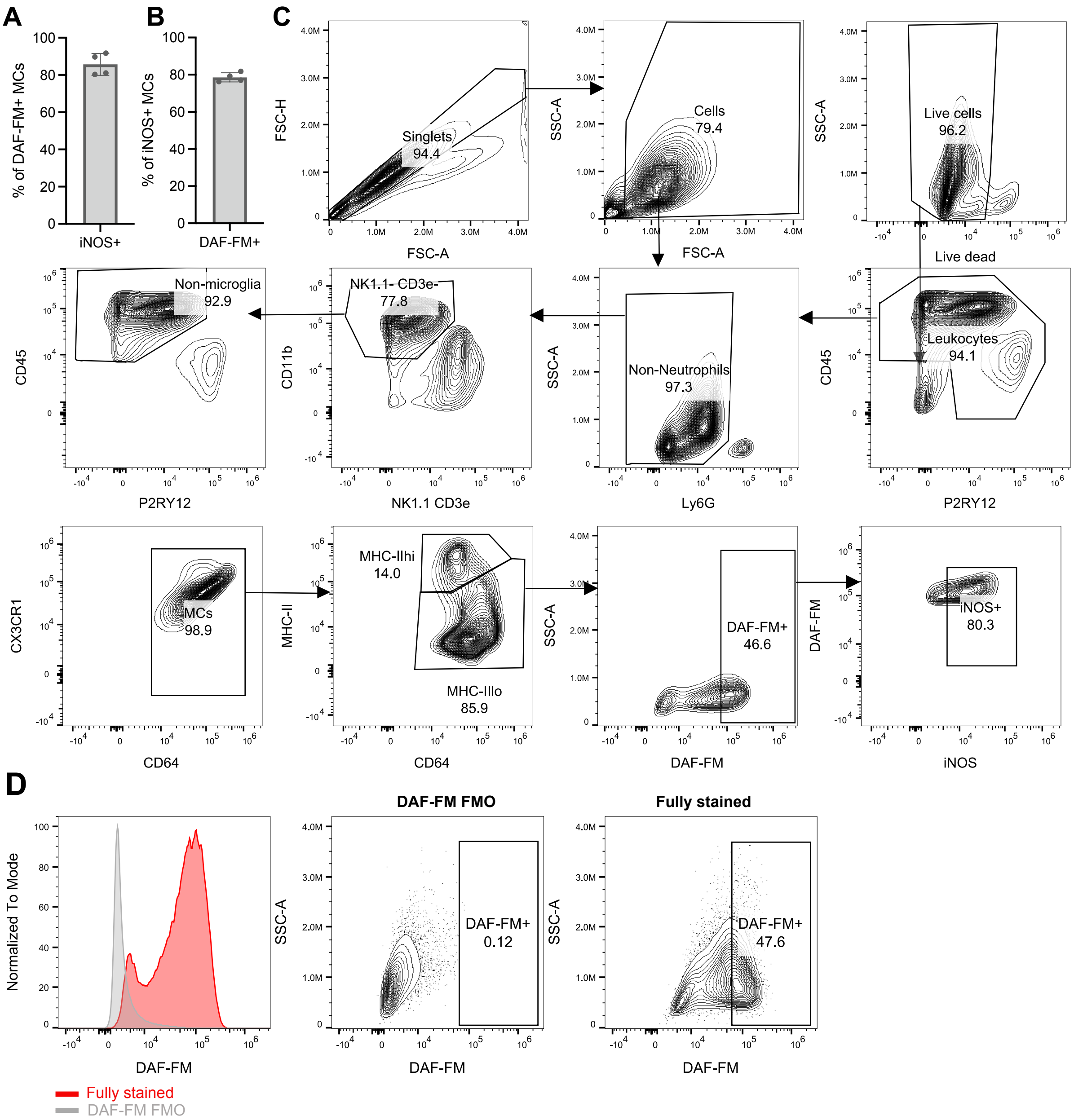

Figure S3. Correlation between iNOS and DAF-FM staining in brain MCs. (**A**) Percent of iNOS+ MCs in the WNV-infected brain that are positive for DAF-FM staining. (**B**) Percent of DAF-FM+ MCs that are positive for iNOS staining. (**C**) Gating strategy for identifying DAF-FM+, iNOS+ MCs, shown on WNV dpi 5. (**D**) DAF-FM gating relative to FMO control, depicted via histogram and dotplot, on MCs derived from the WNV-infected brain on dpi 7. Error bars are representative of mean ± SD.

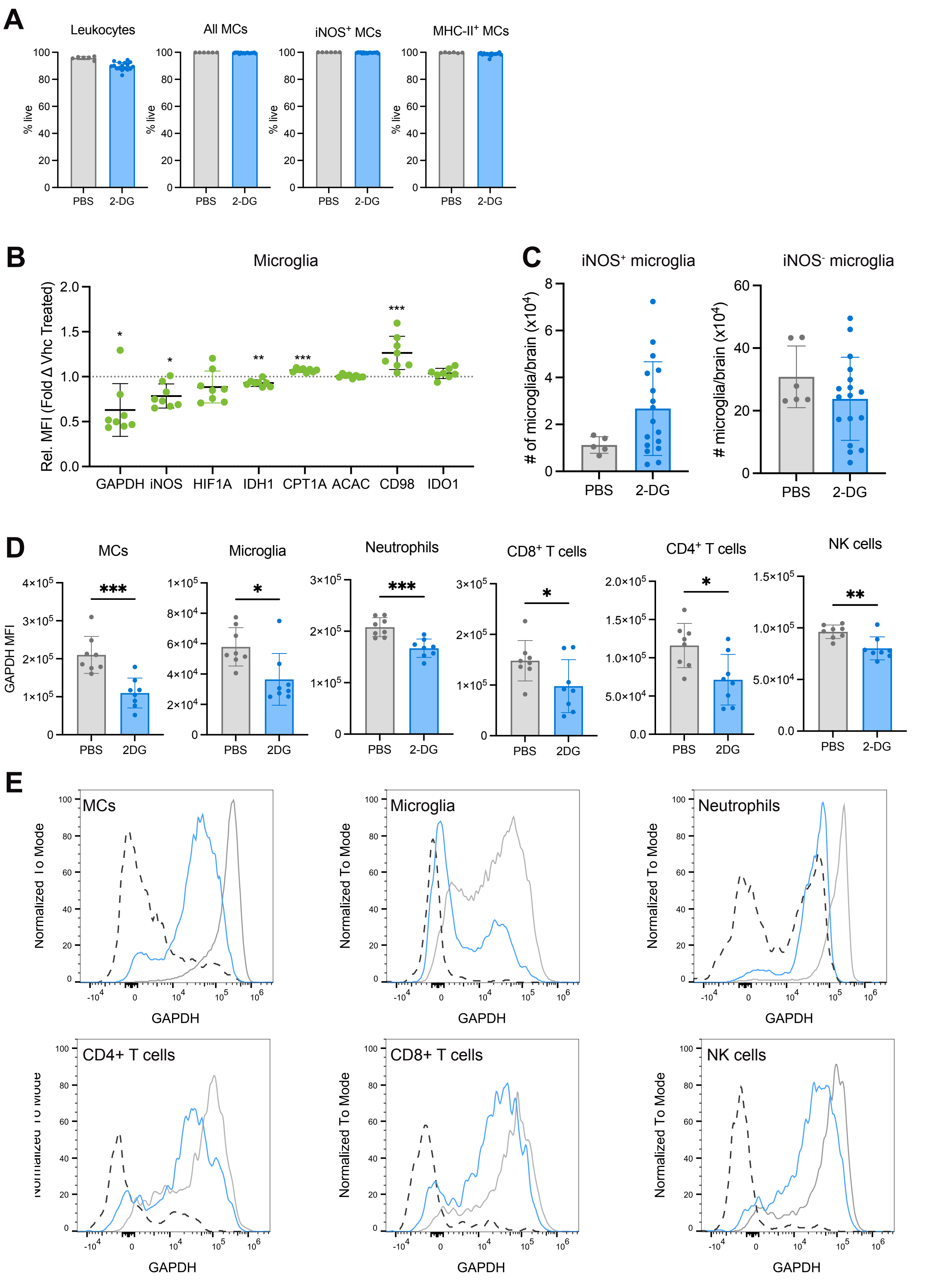

Figure S4. Impact of 2-DG on cell viability, glycolysis markers and inflammatory phenotype of brain leukocytes. (**A**) Percent viability of total leukocytes, total MCs, iNOS+ MCs, and MHC-II+ MCs in 2-DG-treated and PBS-treated mice in the WNV-infected brain. (**B**) Fold-change in median fluorescence intensity of selected metabolic markers in 2-DG-treated mice relative to PBS-treated mice in microglia derived from the WNV-infected brain. (**C**) Number of inflammatory (iNOS+) and non-inflammatory (iNOS-) microglia per brain in PBS and 2-DG-treated mice. (**D**) Median fluorescence intensity of GAPDH across the indicated cell types. (**D**) Histograms depicting the median fluorescence intensity of GAPDH in 2-DG- and PBS-treated mice at 7 dpi, relative to the fluorescence minus one (FMO) control. Data is from one independent experiment with five to 17 mice per group. Statistics calculated by ordinary one-way ANOVA with Tukey's test for multiple comparisons (**B**) or an unpaired t-test (**A, C, D**).* p<0.05, **p<0.01, ***p<0.001. Error bars are representative of mean ± SD.

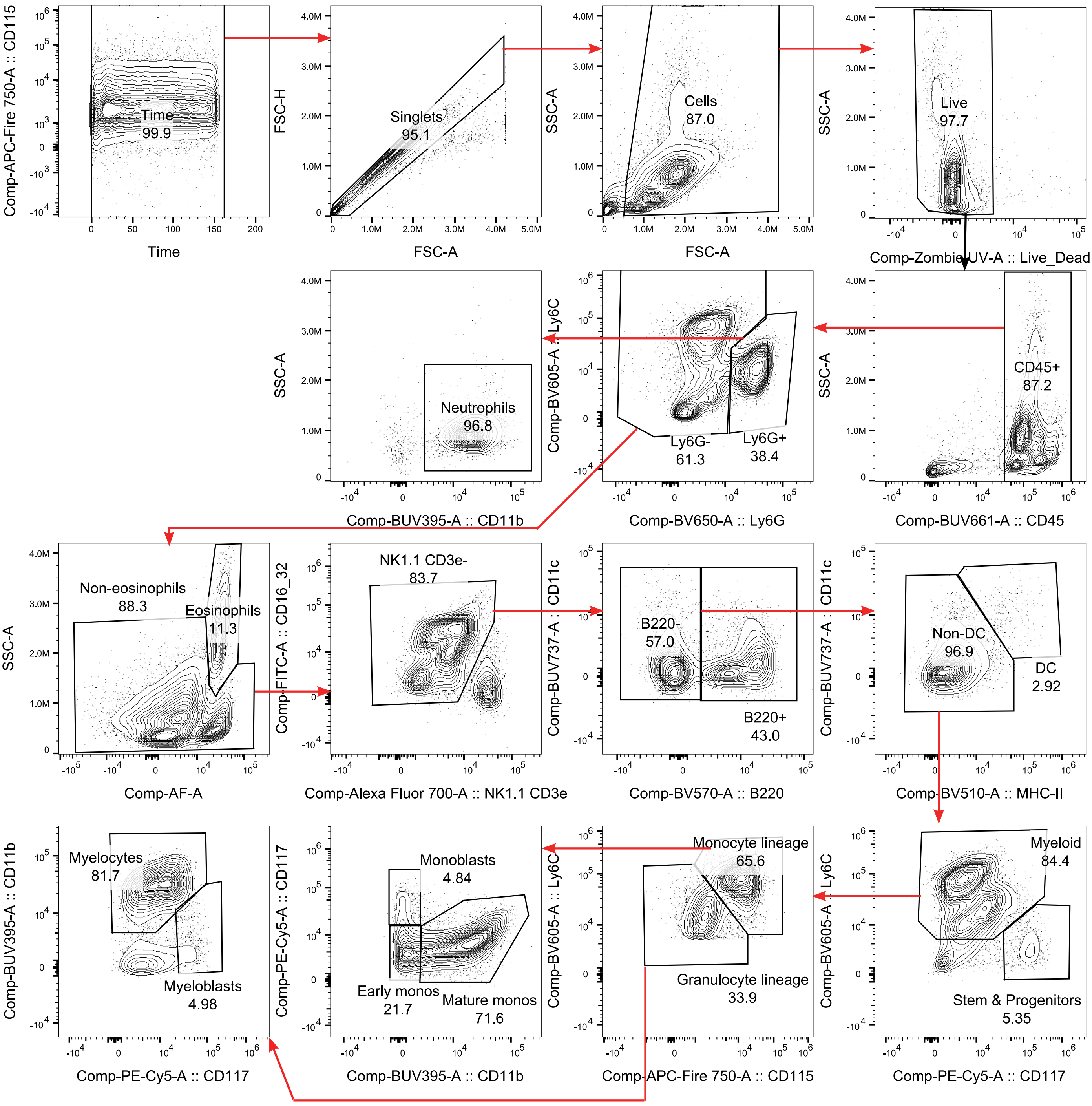

Figure S5. Gating strategy for bone marrow myeloid populations.

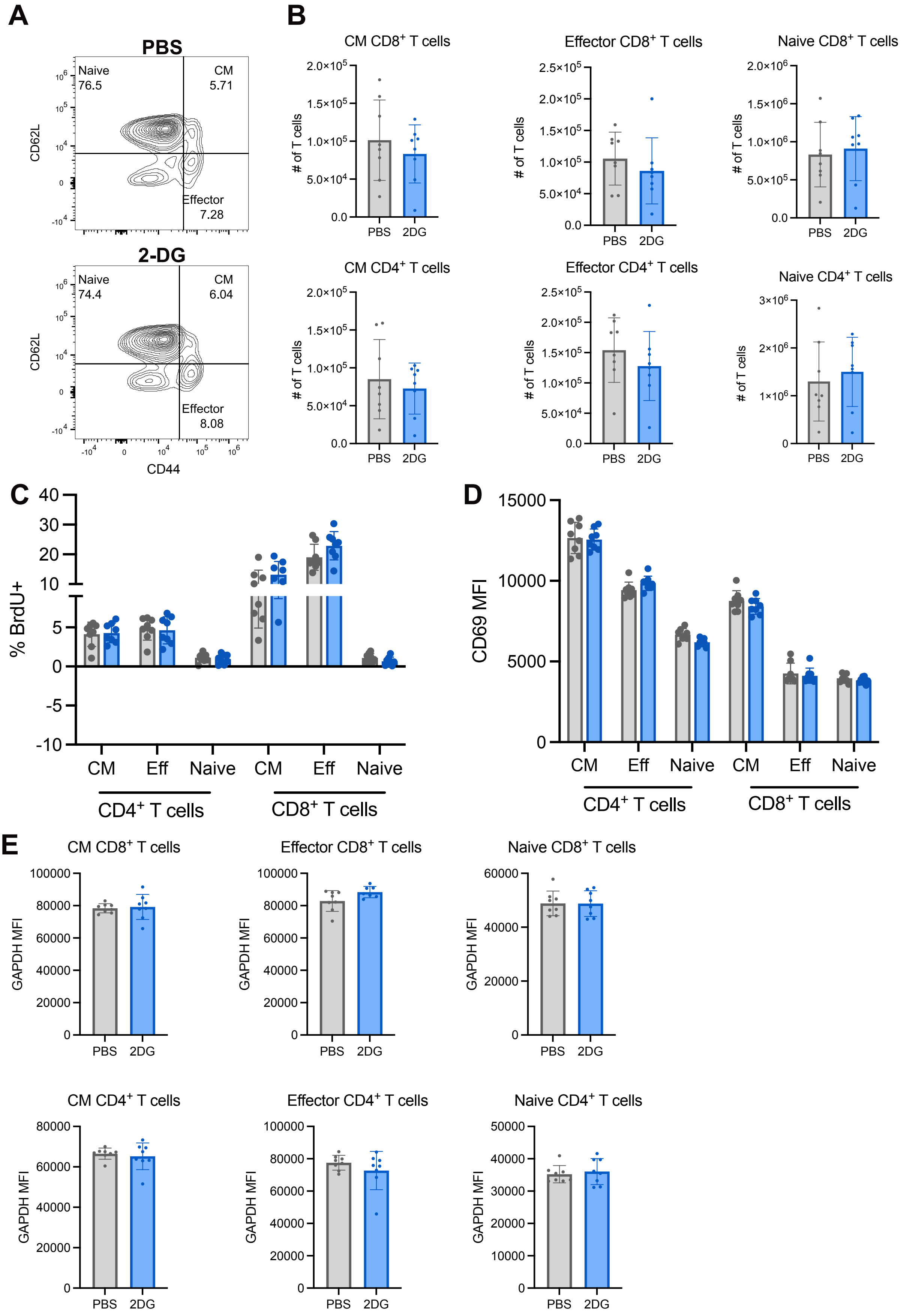

Figure S6. 2-DG treatment from 4-7 dpi does not affect T cell proliferation in draining cervical lymph nodes. (**A**) Contour plot showing gating of naïve, central memory (CM) and effector (eff) CD4+ T cells based on CD62L and CD44 expression. (**B**) Absolute cell numbers of indicated T cell populations per cervical lymph node. (**C, D**) Percentage of BrdU+ T cells (**C**) and their expression of CD69 (**D**) and GAPDH (**E**) in 2-DG- and PBS-treated mice at 7 dpi. Data is from one independent experiment with eight mice per group. Statistics were calculated using an unpaired t-test (**B, E**) or multiple t-tests with two-stage step up method of Bejamini, Krieger, and Yekutieli tests for multiple comparisons (**C, D**). Error bars are representative of mean ± SD. CM, central memory; Eff, effector T cell; Treg, regulatory T cell.

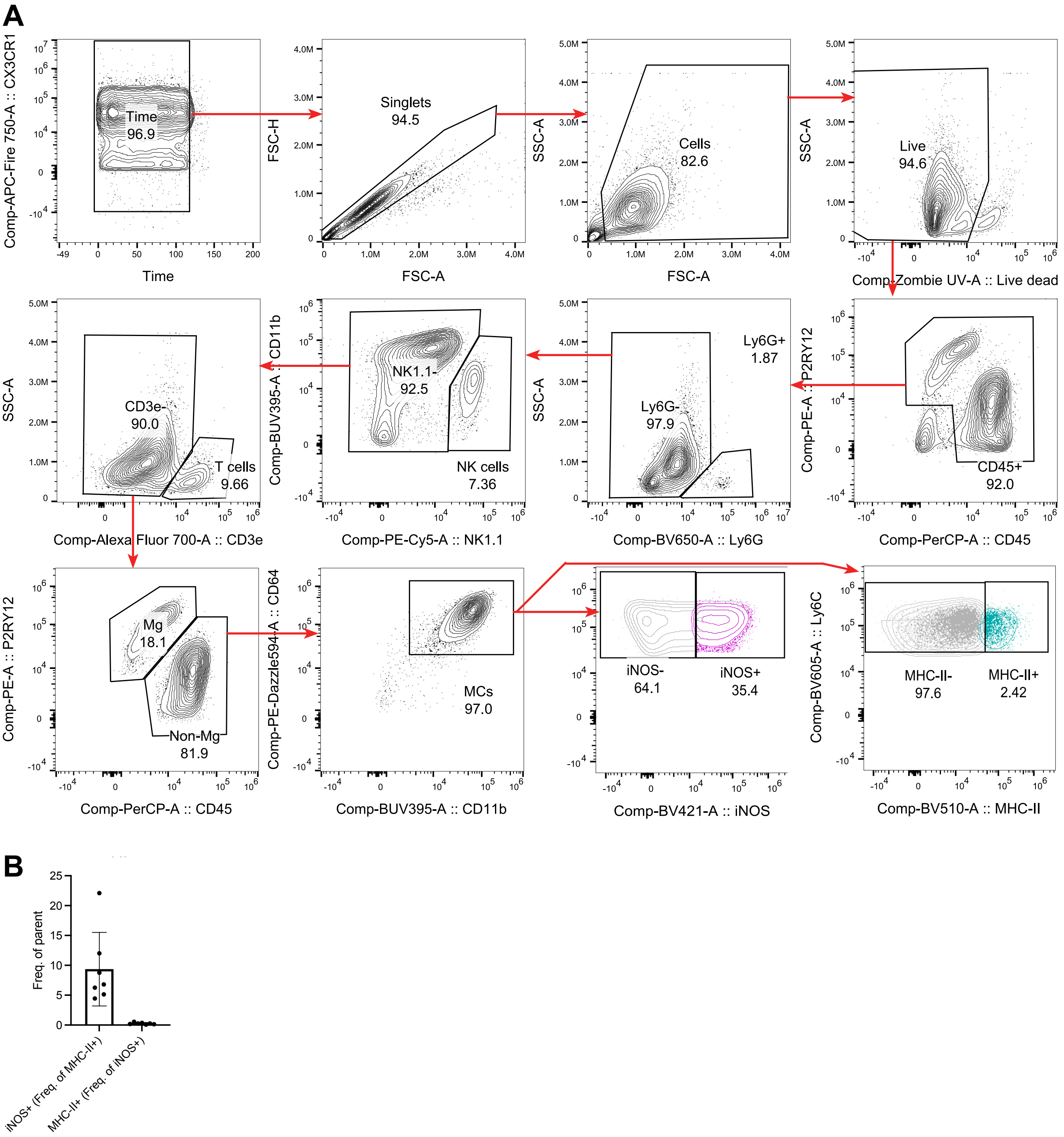

Figure S7. Gating of iNOS+ and MHC-II+ MCs in the WNV-infected brain and glycolysis-associated marker expression in ex vivo M1-stimulated monocytes. (**A**) Gating strategy for iNOS+ and MHC-II+ MC populations in the brain. (**B**) Frequencies of MHC-II+ and iNOS+ MCs expressed as a proportion of each other, demonstrating limited overlap between the two populations. Data is one independent experiment(s) with 3-4 mice per group. Statistics calculated with paired t-test. Error bars are representative of mean ± SD.

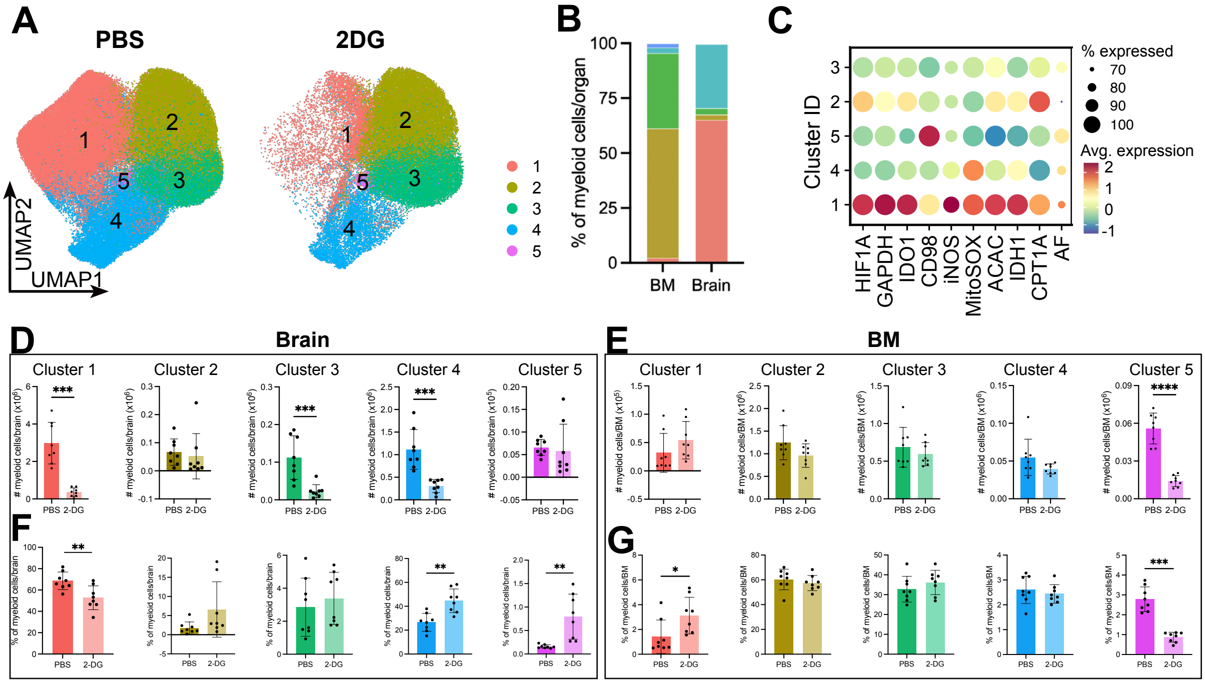

Figure S8. Glycolysis inhibition suppresses pathogenic MC clusters. (**A**) UMAPs pseudocoloured by metabolic cluster ID across PBS- and 2-DG-treated groups. UMAP represents combined myeloid cells from the BM and brain at 7 dpi. (**B**) Frequencies of MHC-II+ and iNOS+ MCs expressed as a proportion of each other, demonstrating limited overlap between the two populations. (**C**) Dot plot heatmap depicting the MFI of each metabolic marker across the identified metabolic cluster at 7 dpi. Size of dot represents the percentage of cells in each c luster expressing the metabolic marker. (**D, E**) Number of myeloid cells per brain (**D**) and monocytes per femur (E) in each cluster in PBS- and 2-DG-treated WNV-infected mice at 7 dpi. (**F, G**) Proportion of each metabolic cluster comprising the total myeloid population in the brain (**F**) and BM (**G**) across treatment groups. Data is one independent experiments with 7-8 mice per group. Statistics performed using unpaired t-test. * p<0.05, ** p <0.01, *** p<0.001, **** p <0.0001. Error bars are representative of mean ± SD.

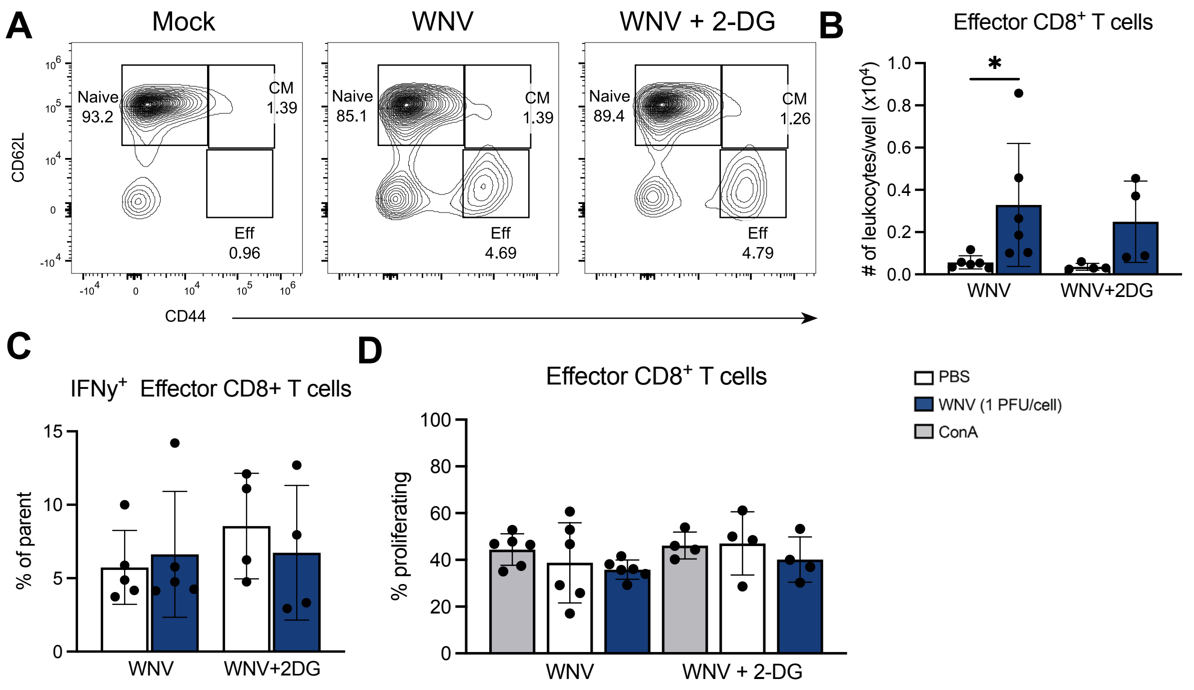

Figure S9. 2-DG treatment does not affect CD8+ T cell functionality from draining cervical lymph nodes. Whole cervical draining lymph nodes were stimulated with WNV or PBS for 1 hour, prior to being cultured with either PBS or Con A for 72 hours, as described in Figure 6. (**A**) Contour plots showing gating of naïve, central memory (CM) and effector (eff) CD8+ T cells based on CD62L and CD44 expression across groups stimulated with WNV. (**B**) Number of effector CD8+ T cells per well of cultured lymph nodes. (**C, D**) Percent of IFN-γ+ (**C**) and proliferating (**D**) effector CD8+ T cells. Data is from one independent experiment with eight mice per group. Statistics calculated by RM one-way ANOVA with Tukey's test for multiple comparisons. * $p<0.05$, ** $p<0.01$. Error bars are representative of mean ± SD.

Supplementary Table 1. Antibodies and reagents used for flow cytometry staining

Antibody	Company	Catalogue	Concentration
**Surface stain antibodies**			
Anti-CD11b (clone M1/70) – BUV395	BD	Cat # 563553	1:100
Anti-CD11b (clone M1/70) – PE-Cy7	Biolegend	Cat # 101216	1:100
Anti-CD45 (clone 30-F11) – BUV661	BD	Cat # 612975	1:100
Anti-CD45.2 (clone 104) – BV650	Biolegend	Cat # 109835	1:50
Anti-CD11c (clone F10/21A3) – BUV737	BD	Cat # 748723	1:100
Anti-CD98 (clone H202-141) – BUV615	BD	Cat # 752360	1:100
Anti-CD8a (clone 53-6.7) – BUV805	BD	Cat # 612898	1:100 (Brain)<br>1:200 (BM, LN)
Anti-MHC-II (clone MP6-XT22) – BV510	Biolegend	Cat # 506339	1:200
Anti-MHC-II (M5/114.15.2) – BV480	BD	Cat # 566088	1:200
Anti-Ly6C (clone HK1.4) – BV605	Biolegend	Cat # 128036	1:100
Anti-Ly6G (clone 1A8) – BV650	Biolegend	Cat # 127641	1:200
Anti-Ly6G (clone 1A8) – FITC	Biolegend	Cat # 127606	1:200
Anti-CD4 (clone GK1.5) – BV750	Biolegend	Cat # 100467	1:100
Anti-CD4 (clone RM4-5) – BV570	Biolegend	Cat #100542	1:100 (Brain)<br>1:200 (BM, LN)
Anti-B220 (clone RA3-6B2) – BV570	Biolegend	Cat # 100542	1:100
Anti-B220 (clone RA3-6B2) – PE-Cy5	Biolegend	Cat #103210	1:100 (Brain)<br>1:200 (BM, LN, spleen)
Anti-NK1.1 (clone PK136) – PE-Cy5	Biolegend	Cat # 108716	1:100 (Brain)<br>1:200 (BM, LN, spleen)
Anti-CD64 (clone X54-5/7.1) – PE/Dazzle594	Biolegend	Cat # 139319	1:200
Anti-CD64 (clone X54-5/7.1) – PE-Cy7	Biolegend	Cat # 139313	1:200
Anti-CD3e (clone 500A2) – AF700	BD	Cat # 557984	1:100 (Brain)<br>1:200 (BM, LN, spleen)
Anti-CD3e (clone 145-2C11) – PE-Cy5	Biolegend	Cat # 100310	1:100 (Brain)<br>1:200 (BM, LN, spleen)
Anti-CX3CR1 (clone SA011F11) – APC/Fire750	Biolegend	Cat # 149040	1:100
Anti-CX3CR1 (clone SA011F11) – BV421	Biolegend	Cat # 149023	1:100
Anti-CD44 (clone IM7) – FITC	Biolegend	Cat # 103006	1:100
Anti-CD44 (clone IM7) – BV650	Biolegend	Cat # 103049	1:100
Anti-P2RY12 (clone S16007D) – APC	Biolegend	Cat # 848006	1:100
Anti-CD62L (MEL-14) – APC	Biolegend	Cat # 104412	1:100
Anti-CD62L (MEL-14) – BV605	Biolegend	Cat # 104438	1:100
Anti-CD69 (H1.2F3) – APC/Cy7	Biolegend	Cat # 104526	1:100

Anti-CD69 (clone H1.2F3) - BV786	BD	Cat # 564683	1:100
Anti-CD48 (HM48-1) – BUV563	BD	Cat # 741258	1:100
Anti-CD48 (clone HM48-1) – APC	Biolegend	Cat # 103412	1:200
Anti-CD48 (clone HM48-1) – APC/Cy7	Biolegend	Cat # 103432	1:200
Anti-CD34 (clone RAM34) – BV421	BD	Cat # 562608	1:100
Anti-Sca-1 (clone D7) – BV711	Biolegend	Cat # 108131	1:200
Anti-CD117 (clone 2B8) – PE-Cy5	Biolegend	Cat # 105810	1:100
Anti-CD117 (clone 2B8) – BV421	Biolegend	Cat # 105828	1:100
Anti-CD115 (clone AFS98) – APC/Fire750	Biolegend	Cat # 135535	1:100
Anti-CD115 (clone AFS98) – PE	Biolegend	Cat #135506	1:100
Anti-CD16/32 (clone 2.4G2) – FITC	Biolegend	Cat # 553144	1:100
Anti-CD16/32 (clone 93) – purified	Biolegend	Cat # 101301	1:100
Anti-CD25 (clone PC61) – PE-Cy7	Biolegend	Cat # 102016	1:200
Anti-CD49d (clone R1-2) – PE	Biolegend	Cat # 103607	1:100
**Intracellular and intranuclear antibodies**			
Anti-iNOS (clone CXNFT) – BV421	Invitrogen	Cat # 404-5920-82	1:100
Anti-GAPDH (clone W17079A) – AF488	Biolegend	Cat # 607905	1:100
Anti-IDH1 (clone RMab 3) - PE	BD	Cat # 567019	1:80
Anti-HIF1α (clone 241812) – APC	R&D systems	Cat # IC1935A	1:100
Anti-IDO1 (clone 2E2) – AF647	Biolegend	Cat # 654003	1:100
Anti-Cytochrome C (clone 6H2.B4) – AF647	Biolegend	Cat # 612310	1:100
Anti-CPT1α (clone 8F6AE9) – Purified	Abcam	Cat # ab128568	1:100
Anti-ACAC– Purified	Abcam	Cat # ab72046	1:50, 1:100
Anti-rabbit IgG – DyLight800	Invitrogen	Cat # **SA5-10036**	1:50
Anti-rabbit IgG (clone Poly4064) – Dylight594 (Secondary Ab for ACAC)	Biolegend	Cat # 406418	1:300
Anti-mouse IgG2b (clone RMG2b-1) – PE-Cy7 (Secondary Ab for CPT1A)	Biolegend	Cat # 406713	1:100
Anti-FoxP3 (MF-14) – PE	Biolegend	Cat # 126404	1:100
Anti-IFNy (XMG1.2) – APC	Biolegend	Cat # 505810	1:100
BrdU – APC	BD	Cat # 51-23619L	1:100
**Isotype controls**			
Anti-Rat IgG2a, k – BUV615	BD	Cat # 751544	1:100
Anti-Rat IgG2a, k – BV421	ThermoFisher Scientific	Cat # 404-4321-81	1:100
Anti-Rat IgG2a, k – AF488	Biolegend	Cat # 400525	1:100
Anti-Mouse IgG1, k – PE	Biolegend	Cat # 400111	1:32
Anti-Mouse IgG2b, k – purified	Biolegend	Cat # 401201	1:100
Anti-Mouse IgG1, k – APC	Biolegend	Cat # 400121	1:100
Anti-Mouse IgG1, k – AF647	Biolegend	Cat # 400130	1:100
Anti-Rabbit IgG – purified	Biolegend	Cat #026102	1:500
**Other dyes and reagents**			
Zombie UV^{TM} Fixable Viability Kit	Biolegend	Cat # 423107	1:500 (Brain) 1:200 (BM, LN)
MitoSOX Red	ThermoFisher Scientific	Cat # M36008	1:4000
DAF-FM diacetate	Invitrogen	Cat # D23844	1:1000

Supplementary Table 2. **Primer sequences used for qPCR**

Primer	NM	Primer set sequence for F 5'-3'	Primer set sequence for R 5'-3'
Tnf	NM_013693.3	ATGGCCTCCCTCTCATCAGT	GTTTGCTACGACGTGGGCTA
Il6	NM_038165.2	CCTCTCTGCAAGAGACTTCCAT	ASTOTCOTCTOOGGACTTOT
Il1b	NM_008361.4	TGCCACCTTTTGACAGTGATG	TGATGTGCTGCTGCGAGATT
Ifng	NM_008337.4	GCAAAAGGATGGTGACATGA	TTCGCCTTGCTGTTGCTGA
Ccl2	NM_011333.3	CAAGATGATCCCAATGAGTAG	TTGGTGACAAAAACTACAGC
Ccl3	NM_011337	CCATATGGAGCTGACACCCC	GAGCAAAGGCTGCTGGTTTC
Ccl5	NM_013653	TGCTCCAATCTTGCAGTCGT	GCAAGCAATGACAGGGAAGC
Ccl7	NM_013654	CTCTCTCACTCTCTTTCTCC	TCTGTAGCTCTTGAGATTCC
Cxcl10	NM_021274	AAAAAGGTCTAAAAGGGCTC	AATTAGGACTAGCCATCCAC
Cxcl16	NM_023158.6	CCATTCTTTATCAGGTTCCAG	CTTGAGGCAAATGTTTTTGG
